# Different configurations of SARS-CoV-2 spike protein delivered by integrase-defective lentiviral vectors induce persistent functional immune responses, characterized by distinct immunogenicity profiles

**DOI:** 10.3389/fimmu.2023.1147953

**Published:** 2023-04-05

**Authors:** Martina Borghi, Alessandra Gallinaro, Maria Franca Pirillo, Andrea Canitano, Zuleika Michelini, Maria Laura De Angelis, Serena Cecchetti, Antonella Tinari, Chiara Falce, Sabrina Mariotti, Antonio Capocefalo, Maria Vincenza Chiantore, Angelo Iacobino, Antonio Di Virgilio, Marit J. van Gils, Rogier W. Sanders, Alessandra Lo Presti, Roberto Nisini, Donatella Negri, Andrea Cara

**Affiliations:** ^1^ Department of Infectious Diseases, Istituto Superiore di Sanità, Rome, Italy; ^2^ National Center for Global Health, Istituto Superiore di Sanità, Rome, Italy; ^3^ Department of Oncology and Molecular Medicine, Istituto Superiore di Sanità, Rome, Italy; ^4^ Confocal Microscopy Unit, Core Facilities, Istituto Superiore di Sanità, Rome, Italy; ^5^ Center for Gender Medicine, Istituto Superiore di Sanità, Rome, Italy; ^6^ Department of Veterinary Public Health & Food Safety, Istituto Superiore di Sanità, Rome, Italy; ^7^ Center for Animal Research and Welfare, Istituto Superiore di Sanità, Rome, Italy; ^8^ Department of Medical Microbiology and Infection Prevention, Amsterdam UMC, University of Amsterdam, Amsterdam Institute for Infection and Immunity, Amsterdam, Netherlands

**Keywords:** lentiviral vector (LV), IDLV, vaccine, SARS-CoV-2, neutralizing Abs

## Abstract

Several COVID-19 vaccine strategies utilizing new formulations for the induction of neutralizing antibodies (nAbs) and T cell immunity are still under evaluation in preclinical and clinical studies. Here we used Simian Immunodeficiency Virus (SIV)-based integrase defective lentiviral vector (IDLV) delivering different conformations of membrane-tethered Spike protein in the mouse immunogenicity model, with the aim of inducing persistent nAbs against multiple SARS-CoV-2 variants of concern (VoC). Spike modifications included prefusion-stabilizing double proline (2P) substitutions, mutations at the furin cleavage site (FCS), D614G mutation and truncation of the cytoplasmic tail (delta21) of ancestral and Beta (B.1.351) Spike, the latter mutation to markedly improve IDLV membrane-tethering. BALB/c mice were injected once with IDLV delivering the different forms of Spike or the recombinant trimeric Spike protein with 2P substitutions and FCS mutations in association with a squalene-based adjuvant. Anti-receptor binding domain (RBD) binding Abs, nAbs and T cell responses were detected up to six months from a single immunization with escalating doses of vaccines in all mice, but with different levels and kinetics. Results indicated that IDLV delivering the Spike protein with all the combined modifications, outperformed the other candidates in terms of T cell immunity and level of both binding Abs and nAbs soon after the single immunization and persistence over time, showing the best capacity to neutralize all formerly circulating VoC Alpha, Beta, Gamma and Delta. Although present, the lowest response was detected against Omicron variants (BA.1, BA.2 and BA.4/5), suggesting that the magnitude of immune evasion may be related to the higher genetic distance of Omicron as indicated by increased number of amino acid substitutions in Spike acquired during virus evolution.

## Introduction

The severe acute respiratory syndrome coronavirus 2 (SARS-CoV-2) belongs to the Betacoronavirus genus and is the agent causative of coronavirus disease 2019 (COVID-19). COVID-19 outbreak has been declared a pandemic by the World Health Organization (WHO) in March 2020 and soon after has become a global health priority, with over 757 million confirmed infections and more than 6.8 million deaths as of February 2023 (1). As a consequence, a global effort started to develop effective preventative interventions against SARS-CoV-2.

SARS-CoV-2 utilizes the transmembrane homotrimeric Spike glycoprotein to enter into target cells *via* the angiotensin-converting enzyme 2 (ACE2) receptor (2). As a consequence, neutralizing antibodies (nAbs) against the viral Spike protein are an essential component of the protective immune response against SARS-CoV-2 (3). Several effective vaccines delivering Spike, including mRNA, protein subunit, adenoviral vector, and whole-cell inactivated virus, showed efficacy in phase III trials and have received approval for use in many countries (4), and more are under consideration (5). While these vaccination approaches have proved remarkably successful in limiting viral spread and disease, mutations that affect transmission and disease severity have occurred throughout the pandemic. Indeed, the high infection rates and the immune selection pressure induced by the vaccines at a population level have accelerated the development of escape mutants, as demonstrated by the insurgence and spreading of several variants of interest and of concern (VoI and VoC), thus posing a threat to the long-term effectiveness of these vaccines. In particular, VoC have specific mutations in their Spike proteins that have been associated with breakthrough infections, increased transmissibility (6–11) and decreased sensitivity against neutralization by monoclonal Abs (mAbs), sera from vaccinated individuals and convalescent plasma (12–24). Therefore, vaccines against COVID-19 need continuous optimization and updating as a matter of urgency. In addition, the lack of sterilizing immunity and the modest durability of the protective immune responses induced by currently approved vaccines require additional boosters in a relatively short interval of time, decreasing the overall vaccine compliance. As a consequence, persistent and cross-reactive vaccination approaches should be sought to prevent close vaccination cycles, especially in developing countries with a low-resource setting, where the cost and logistics of vaccine campaigns are difficult (25–28).

Integrase-defective lentiviral vectors (IDLVs) offer a safe alternative vaccination approach with features similar to live attenuated virus including sustained antigen expression from the episomal forms of the vector, but in the absence of integration- and replication-competent virus (29–31). IDLVs showed their efficacy to induce high magnitude and long-lasting antigen-specific cellular and humoral immunity in mice, non-human primates (NHPs) and humans (32–36). Importantly, recent reports showed efficacy of non-integrating lentiviral vector against SARS-CoV-2 in mice immunogenicity studies (37, 38). In order to improve immunogenicity, we demonstrated that IDLV can be exploited for delivering immunogens also after pseudotyping of heterologous viral envelope glycoproteins on the vector’s particles, in addition to VSV.G. In particular, pseudotyping of IDLV with influenza virus hemagglutinin (HA) resulted in the induction of an anti-HA nAb response in mice, which was persistent for up to 24 weeks after a single immunization (39). More recently, we demonstrated that truncation of cytoplasmic tail of HIV-Env and SARS-CoV-2 Spike glycoproteins greatly improved the pseudotyping of lentiviral particles (34, 40), leading to persistent immunogenicity in the NHP model of immunization using HIV-Env (34).

Although IDLVs provide strong and durable immune responses, the encoded antigen also dictates the quality and magnitude of those responses, and different design elements of the Spike protein may influence the performance of the immune response after immunization. In this report, we have developed and used Simian Immunodeficiency Virus (SIV)-based IDLV for the delivery of different conformations of Spike (IDLV-CoV2) in the mouse immunogenicity model. In particular, we evaluated the introduction of substitutions to stabilize Spike in the prefusion conformation, including mutations at the furin cleavage site (FCS) and of two consecutive prolines (2P) in the hinge region of S2 portion (2, 41), truncation of the Spike protein cytoplasmic tail to favour Spike membrane-tethering on IDLV particles (40), and the inclusion of the D614G mutation to enhance the exposure of the receptor binding motif (RBM) (42). When combined together, these mutations increased the magnitude of nAbs against the autologous Spike compared to IDLV delivering wild-type Spike and to the subunit vaccine and elicited higher cross-reactive nAbs against all VoC, including Omicron. Importantly, neutralizing activity persisted up to 6 months from a single immunization even at the lowest dose of the IDLV expressing the fully modified Spike, confirming the ability of IDLV to elicit cross-reactive long term functional immunity.

## Materials and methods

### Plasmids construction

A schematic representation of the plasmids, transfer vectors and coding sequences used in this study is shown in **Supplementary Figure 1**. All Spike sequences expressed from lentiviral transfer vectors were obtained after codon optimization and cloning into pUC57 vector (GenScript Biotech, Rijswijk, Netherlands). Spike sequences were removed from pUC57 plasmids with AgeI/SalI restriction sites and cloned into the corresponding restriction sites of pGAE-GFP self-inactivating lentiviral transfer vector plasmid by substituting the GFP coding sequence (43) (**Supplementary Figure 1A**). Plasmid pGAE-Spike encodes the codon optimized full-length wild type SARS-CoV-2 Spike protein open reading frame (ORF) (Wuhan-Hu-1, GenBank: NC_045512.2); plasmid pGAE-S2PF encodes the Spike ORF, stabilized by the introduction of 2 prolines (2P, K986P and V987P) and by functional mutation of RRAR into GSAS at the furin cleavage site (FCS); plasmid pGAE-S2PGC encodes the Spike ORF and contains a 21 amino acid (aa) deletion in the cytoplasmic tail (delta21) with 2P and D614G mutations; plasmid pGAE-betaS2PGC encodes the delta21 codon optimized B.1.351 (beta) Spike ORF with the 2P stabilizing mutation; plasmid pGAE-S2PFGC encodes the delta21 Spike ORF with the 2P, D614G and FCS mutations; plasmids pGAE-JR, pGAE-Luc and pGAE-GFP encode the HIV-1_JR-FL_ gp120 envelope, the luciferase and the GFP ORFs, respectively (44). Plasmids pAdSIVD64V and pAdSIV3+ and are the Integrase defective and Integrase competent packaging vectors, respectively (43). Plasmid phCMV-VSV.G encodes the vesicular stomatitis virus envelope glycoprotein G (VSV.G), used for pseudotyping of lentiviral vector (43).

Expression plasmids encoding wild type and variants of concern (VoC) Spike ORFs used in the pseudovirus neutralization assay were previously described (45, 46) and are depicted in **Supplementary Figure 1B**. Briefly, plasmid pSpike-C3 encodes the wild type (Wuhan-Hu-1) codon optimized SARS-CoV-2 delta21 Spike protein open reading frame (ORF) (40); plasmids pSpike-UKC3, pSpike-SAC3 and pSpike-BRC3 encode the delta21 codon optimized B.1.1.7 (Alpha), B.1.351 (Beta) and P.1 (Gamma) Spike ORFs, respectively, while plasmid pSpike-INC3 encodes the B.1.617.2 (Delta) Spike ORF with a 19 aa deletion in the cytoplasmic tail (45, 46). For construction of plasmid, expressing the delta21 codon optimized B.1.1.529 (Omicron) BA.1, BA.2 and BA.4/5 Spike ORF VoC, a NheI/BamHI fragment of DNA was removed from pUC57-BA.1, pUC57-BA.2 and pUC57-BA.4/5 plasmids (GenScript) and inserted into the pSpike-C3 plasmid at the corresponding restriction sites, to obtain pSpike-BA1C3, pSpike-BA2C3 and pSpike-BA4C3 plasmids. The B.1.1.7 Spike utilized for these studies contains the mutations: del69-70, del144, N501Y, A570D, D614G, P681H, T716I, S982A, D1118H. The B.1.351 Spike utilized for these studies contains the mutations: L18F, D80A, D215G, del242-244, R246I, K417N, E484K, N501Y, D614G, A701V. The P.1 Spike utilized for these studies contains the mutations: L18F, T20N, P26S, D138Y, R190S, K417T, E484K, N501Y, D614G, H655Y, T1027I. The B.1.617.2 Spike utilized for these studies contains the following mutations: T19R, del157-158, L452R, T478K, D614G, P681R, D950N. The BA.1 Spike utilized for these studies contains the mutations: A67V, del69/70, T95I, G142D, del143-145, N211I, del212, ins214EPE, G339D, S371L, S373P, S375F, S477N, T478K, E484A, Q493R, G496S, Q498R, N501Y, Y505H, T547K, D614G, H655Y, N679K, P681H, D796Y, N856K, Q954H, N969K, L981F. The BA.2 Spike utilized for these studies contains the following mutations: T19I, L24S, del25/27, G142D, V213G, G339D, S371F, S373P, S375F, T376A, D405N, R408S, K417N, N440K, S477N, T478K, E484A, Q493R, Q498R, N501Y, Y505H, D614G, H655Y, N679K, P681H, N764K, D796Y, Q954H, N969K. The BA.4/5 Spike utilized for these studies contains the following mutations: T19I, L24S, del25/27, del69/70, G142D, V213G, G339D, S371F, S373P, S375F, T376A, D405N, R408S, K417N, N440K, L452R, S477N, T478K, E484A, F486V, Q498R, N501Y, Y505H, D614G, H655Y, N679K, P681H, N764K, D796Y, Q954H, N969K.

### Production of integrase defective lentiviral vectors for immunization

293T Lenti-X cells (Clontech, Mountain View, CA, USA) were utilized for the production of SIV-based IDLV by transient transfection, as described (34). Cells were kept in Dulbecco’s modified Eagles medium, with high glucose 4.5 g/L (Gibco, Life Technologies Italia, Monza, Italy) and were supplemented with 100 units/ml penicillin/streptomycin (Gibco) and 10% fetal calf serum (Corning, Mediatech, Manassas, VA, USA). Cells (3.5x10^6^ cells) were plated on 10 cm Petri dishes (Corning Incorporated-Life Sciences, Oneonta, NY, USA) and transfected with each self-inactivating lentiviral transfer vector plasmid pGAE expressing the different Spike(s), RBD or HIV-1_JR-FL_ gp120 envelope, the packaging plasmid integrase-defective pAdSIVD64V and the VSV.G pseudotyping plasmid phCMV-VSV.G, to allow entry into target cells *in vitro* and *in vivo*, with the CalPhos™ Mammalian Transfection Kit (Clontech Laboratories, Inc, Mountain View, CA, USA) by following the recommendations of the manufacture using a plasmid ratio of 1:2:1 (transfer vector: packaging plasmid: VSV.G plasmid). Forty-eight hours post transfections, the supernatants of the cultures containing the IDLV pseudotyped with different conformation of Spike (IDLV-CoV2) (IDLV-Spike, IDLV-S-2PF, IDLV-S-2PGC, IDLV-betaS-2PGC and IDLV-S-2PFGC) and IDLV-JR were collected, filtered with a 0.45 μm pore size filter (Millipore Corporation, Billerica, MA, USA) and concentrated by ultracentrifugation for 2.5 h at 65,000 × g using a 20% sucrose cushion. Vector particles were dissolved in 1× phosphate buffered saline (PBS, Gibco) and stored at −80°C. Each stock of IDLV was titered using the reverse transcriptase (RT) activity assay (34).

### Production of lentiviral vectors expressing Luciferase and pseudotyped with Spike variants

Spike variants pseudotyped lentiviral vectors expressing Luciferase (LV-Luc) were generated by transient transfection of 293T Lenti-X cells as previously described (40, 45, 46). In brief, 293T Lenti-X cells (3.5x10^6^ cells) were plated on 10 cm Petri dishes (Corning) and transfected with the lentiviral transfer vector plasmid pGAE-Luc expressing the luciferase coding sequence (44), the packaging plasmid pAd-SIV3+ and each of the pseudotyping plasmid expressing Spike protein described above (**Supplementary Figure 1B** **)** or control VSV.G (phCMV-VSV.G) utilizing the JetPrime transfection kit (Polyplus Transfection, Illkirch, France) using a plasmid ratio of 1:2:1 (transfer vector: packaging plasmid: Spike/VSV.G plasmid). Forty-eight hours post transfection, the supernatants containing the LV-Luc pseudoviruses were collected, filtered with a 0.45 μm pore size filter (Millipore) and stored in 0.5 mL aliquotes at -80°C.

### Western blot

Concentrated preparations of IDLV were lysed in SDS loading buffer and resolved on SDS-PAGE (10% polyacrylamide) under reducing conditions. Nitrocellulose membrane was used to transfer gels with a Trans-Blot Turbo System (Bio-Rad Laboratories, Hercules, CA, USA). Filters were saturated with 5% nonfat dry milk dissolved in TBST (TBS with 0.1% Tween 20) for 1 hour and further incubated with anti-S2 polyclonal antibody (dilution 1:2000. Cat: 40590-T62, Sino Biological, Beijing, China), anti-S1 polyclonal antibody (dilution 1:1000. Cat: 40150-T62-COV2, Sino Biological, Beijing, China) or anti-HIV-1 SF2 p24 polyclonal Ab (dilution 1:4000. ARP-4250, NIH HIV Reagent Program, Manassas, VA, USA), followed by anti-rabbit horseradish peroxidase (HRP)-conjugated Ab (dilution 1:3000; Bio-Rad Laboratories, Hercules, CA, USA). Recombinant trimeric Spike with 2P and FCS mutations (47), RBD (45) and SIVmac239 p27 (ARP-13446, NIH HIV Reagent Program) proteins were used as positive controls, whereas preparations from unrelated LV or IDLVs were used as negative controls. WesternBright ECL detection system (Advansta, San Jose, CA, USA) was used as chemiluminescent substrate. Images were acquired and elaborated by ChemiDoc XRS+ System (Bio-Rad Laboratories, Hercules, CA, USA).

### Flow cytometry

293T Lenti-X cells (3x10^5^/well) were plated in 6-well plate and transfected with 1 µg of Spike-expressing plasmids or phCMV-VSV.G as a negative control using the CalPhos™ Mammalian Transfection Kit (Clontech). Forty-eight hours after transfection, cells were detached, counted and stained either with anti-S2 commercial antibody (Cat: 40590-T62, Sino Biological; 1:3000) followed by donkey anti-rabbit PE (Biolegend, San Diego, CA, USA; 4 µg/ml) or human monoclonal antibodies COVA2-15, COVA1-16, COVA1-18, COVA1-21 (47) (1 µg/ml) or CR3022 (Cat: ab273073, Abcam, Cambridge, UK; 5 µg/ml), followed by Goat anti-human IgG secondary AlexaFluor647 (Cat: 109-605-003, Jackson ImmunoResearch, 5 μg/ml). The expression of Spike was analyzed by flow cytometry utilizing a FACSCalibur (BD Biosciences, Milan, Italy), and the results were analyzed with Kaluza software (Beckman Coulter, Fullerton, CA, USA).

### Transmission electron microscopy analysis

293T Lenti-X cells (3.5x10^6^ cells) were transfected on 10 cm Petri dishes (Corning) to produce each IDLV-CoV2 as described above. At 48 hrs post transfection, cells were stained with anti-Spike COVA2-15 mAb (47), with the exception of IDLV producing beta Spike, which was stained with COVA1-16 mAb (47), followed by Goat Anti-Human IgG H and L (10 nm Gold) used as a secondary Ab (Abcam, Cambridge, UK). After staining, cells were fixed with 2.5% glutaraldehyde in cacodylate buffer 0.1 M, pH 7.2. Fixed cells were washed and post-fixed in 1% OsO4 using the same glutaraldehyde/cacodylate buffer. Fixed specimens were dehydrated by using a graded series of ethanol solutions and then embedded using an Agar 100 resin (Agar Scientific, Essex, UK). Ultrathin sections were placed on 200-mesh copper grids and then stained with lead citrate and uranyl acetate. Sections were analysed by using a Philips 208S transmission electron microscopy (TEM) at 100 kV.

### Confocal laser scanner microscopy

293T Lenti-X cells (2.5x10^4^/well) were plated onto L-polylysine (Sigma) treated 12-mm cover glasses inserted in 24-well microplates and then transduced with 5 MOI of each LV expressing Spikes used for immunization. At 48 hours post-transduction, the cells were washed and stained with anti-Spike COVA2-15 or COVA1-16 (47) mAbs followed by AlexaFluor 488 Goat anti-human IgG as a secondary Ab (Jackson ImmunoResearch, 0.4 µg/sample). The coverslips were extensively rinsed, fixed with cold methanol and then placed on the microscope slides using Vectashield antifade mounting medium, containing DAPI (Vector Labs, Burlingame, CA, USA).

Observations by confocal laser scanner mycroscopy (CLSM) were performed using a Zeiss LSM980 apparatus (Zeiss, Oberkochen, Germany), fitted with a Plan-Apochromat 63x/1.4 NA using the appropriate spectral laser lines. Acquisition and processing of images was performed by using Adobe Photoshop CS5 software programs (Adobe Systems, San Jose, CA, USA) and Zen Blue edition 3.3 (Zeiss). Cells which were stained with the secondary antibody were used to set up parameters of acquisition. Several fields (including >200 cells) were evaluated for each labeling condition, and shown are representative results.

### Mouse immunization protocol

BALB/c mice, obtained from Charles River (Charles River, Calco, Como, Italy), were housed in the animal facility at the Istituto Superiore di Sanità (ISS, Rome, Italy) under specific pathogen-free conditions. All procedures have been performed in accordance with Italian legislation and European Union guidelines for animal care. All studies have been reviewed by the Service for Animal Welfare at ISS and authorized by the Italian Ministry of Healthy (Authorization n. 731/2020-PR, 21/7/20, prot. D9997.107). Mice (six animals per group) were immunized once intramuscularly (i.m.) with escalating doses (1.56x10^6^ - 6.25x10^6^ - 25x10^6^ RT units/mice) of IDLV-CoV2 or Spike protein (10 and 1 µg/mouse) adjuvanted with Addavax (*In vivo*gen Europe). Naïve and mice injected with the highest dose of IDLV-JR (mock), expressing the HIV-1_JR-FL_ gp120 envelope (32), were used as negative controls. Retro orbital sampling of blood was carried out prior to immunization and at monthly intervals with glass Pasteur pipettes and sera were collected and stored at -80°C. Six months after the immunization, all mice were sacrificed and spleen harvested and processed for the analysis of T cell responses, as previously described (32). Briefly, single-cell suspensions of splenocytes were obtained after mechanical disruption of spleens in the presence of 3 mL of ACK followed by passage through cell strainers (Corning, Merk Life Science S.r.l., Italy). Splenocytes were then washed using RPMI 1640 medium supplemented with 1% penicillin/streptomycin, 2 mM L-glutamine, 10% FBS, and 50 mM 2-mercaptoethanol (complete medium). Splenocytes were centrifuged at 1500 rpm for 10 min at 4°C and then resuspended in complete medium, counted and stored in liquid Nitrogen.

### ELISA

SARS-CoV-2 recombinant RBD (rRBD) protein produced in HEK293T cells (45) was used for coating 96 well plates (Greiner bio-one, Frickenhausen, Germany) using 0.1 µg/well of rRBD protein overnight at 4°C. Plates were washed and blocking was performed for 2 h by using 1% BSA (Sigma Chemicals) in 200 µL of PBS. Plasma from individual mice were added to wells in duplicate of serial dilutions and further incubated for 2 h at room temperature. After washing, the plates were incubated with biotin-conjugated goat anti-mouse IgG (Southern Biotech, Birmingham, AL, USA) for 2 h at room temperature. The plates were washed and streptavidin-conjugated horse radish peroxidase (HRP) (AnaSpec, Fremont, CA, USA) was added to the plates for 30 min at room temperature. The reaction was incubated with 3.3,5.5-tetramethylbenzidine substrate (SurModics BioFX, Edina, MN, USA) and then blocked using of H2SO4 1 M (50 µL). Endpoint titers were calculated as the reciprocal of the highest dilution with an absorbance value equal at least three times the values from naïve mice. Results were expressed as mean titer ± standard error of the mean (SEM) for each group.

### Pseudovirus titration and neutralization assay

Preparations of LV-Luc pseudoviruses (LV-Luc/Spike-C3, LV-Luc/Spike-SAC3, LV-Luc/Spike-UKC3, LV-Luc/SpikeBRC3, LV-Luc/SpikeINC3, LV-Luc/SpikeOMC3 (BA.1, BA.2, BA.4/5) and LV-Luc/VSV.G) were titered in Vero E6 cells (Cercopithecus aethiops derived epithelial kidney, ATCC C1008). Cells (2.2x10^4^ cells/well) were plated in 96-well plates (Viewplate, PerkinElmer). After forty-eight hours, luciferase expression was measured with a Varioskan luminometer (TermoFisher) by using the britelite plus Reporter Gene Assay System (PerkinElmer). In the neutralization assay were used dilutions providing 2x10^5^ relative light units (RLU). In brief, serum serial 2-fold dilutions starting from 1:80 were incubated in duplicate with the LV-Luc pseudotypes at 37°C for 30 min in 96-deep well plates (Resnova, Roma, Italy). The mixture was then added to Vero E6 cells seeded in a 96-well Isoplate (Perkin Elmer, Groningen, Netherlands) at a density of 2.2x10^4^ cells/well. Cell only and virus -only controls were included. After forty-eight hours, luciferase expression was measured as above by using the britelite plus Reporter Gene Assay System (Perkin Elmer). RLU numbers were transformed into percentage neutralization values, and relative to virus-only controls. Results were expressed as the inhibitory concentration (ID) 50, which corresponds to the dilution of serum providing 50% inhibition of the infection (corresponding to neutralization), compared to the virus-only control wells. ID50 was calculated with a linear interpolation method (40).

### SARS-CoV-2 microneutralization assay

The SARS-CoV-2 isolate (GenBank: MT066156.1; Cat: NR-52284; SARS-Related Coronavirus 2, Isolate Italy-INMI1; BEI Resources, Manassas, VA, USA) was propagated by inoculation of 70% confluent Vero E6 cells in 175 cm^2^ cell culture flasks. The cells were kept in Dulbecco’s modified Eagles medium, high glucose 4.5 g/L (Gibco) supplemented with 2% fetal calf serum (Corning), 100 units/ml penicillin/streptomycin (Gibco), 1 mM sodium pyruvate (Gibco), and 1x non-essential amino acids (Gibco) (48). Briefly, cells were maintained at 37°C and supernatant collected at 72 hrs post inoculation, when a strong cytopathic effect (CPE) was observed. Supernatants were collected, clarified to remove cellular debris, aliquoted, and stored at -80°C. The tissue culture infectious dose 50 (TCID_50_) was determined on flat-bottom 96‐well culture plates of Vero E6 cells (2.2x10^3^ cells/well) by end-point titration of serial 1 log dilutions (from 1 log to 11 log) of viral stocks. Cells were further incubated for 5 days and monitored daily for CPE. TCID_50_/ml of the SARS-CoV-2 was estimated as 7.5x10^6^ TCID50/ml employing the Spearman-Karber method (49). Biosafety level 3 facilities were used for all viral manipulations.

The virus neutralization (VN) assay was executed as reported (50) using heat-inactivated samples (30 min at 56°C). Sera were mixed with an equal volume of 100 TCID_50_ SARS-CoV-2 and incubated for 1 hr at room temperature. A 100 µL of virus–serum mixture was subsequently added to 96-well plates containing a Vero E6 cell monolayer. After 4 days of incubation at 37°C and 5% CO_2_ in humidified atmosphere, the plates were inspected by an inverted optical microscope for presence/absence of CPE. The highest serum dilution that protected from CPE more than 50% of the cells was used to calculate the VN titer, which was expressed as the reciprocal of the highest serum dilution protecting from CPE.

### IFNγ/IL-5 FluoroSpot assay

The assay was performed using Mabtech reagents and protocol (FluoroSpot Plus, Mabtech AB, Sweden). Briefly, 96-well plates were coated with anti-IFNγ and anti-IL-5 antibodies and then blocked with complete medium. Splenocytes were seeded at a density of 3x10^5^/well and stimulated overnight with 1 µg/mL of SARS-CoV-2 Wuhan Spike peptide pool of 15-mer sequences with 11 amino acids overlap (PepTivator^®^ SARS-CoV-2 Prot_S complete, Miltenyi Biotec, Bologna, Italy), as specific stimulation. Positive control included Concanavalin A (5 µg/mL, Sigma Chemicals), while the complete medium was utilized as a negative control. Wells were washed and incubated with anti-IFNγ and anti-IL-5 detection antibodies. After washes, wells were incubated with fluorophore-conjugates for 60 min and after washes a fluorescence enhancer was added. Spot Forming Cells (SFC) were counted using a FluoroSpot reader (AID iSpot, AID GmbH, Strassberg, Germany) and results were expressed as SFC/10^6^ cells. The number of SFC counted in the wells treated with the medium (background) was subtracted from the number of SFC counted in the wells treated with the specific peptides. Samples were recorded positive when the number was equivalent to at least 50 specific SFC/10^6^ cells and was two-fold higher than the background values.

### Phylogenetic analysis

A total of 59 SARS-CoV-2 sequences were used for this study, 51 of which were downloaded from the GISAID database (51) and 8 were SARS-CoV-2 Spike protein sequences above described (**Supplementary Table 1**). The sequences were cut respect to the Spike protein positions of the Wuhan-Hu-1 Accession Number: NC_045512. The translation from nucleotide sequences to protein sequences was performed through Expansy tool (52). The sequence alignments were done using MAFFT v7 (53) with the Galaxy platform (54) and edited manually by utilizing BioEdit v. 7.2.6.1 (55). The maximum likelihood phylogenetic trees were estimated with the software PhyML version 3.0 (56). The statistical significance in the tree has been evaluated by using the Fast likelihood-based method, aLRT-SH like branch support. The Smart Model Selection software (57) was used to find the best-fit substitution models for protein alignments. In addition to the spike protein, three further protein alignments corresponding to the S1, S2, RBD portions were generated.

### Genetic distance analyses

SARS-CoV-2 protein sequences of the S1, S2, RBD alignments were grouped according to the variant to which they belong and their genetic distances were calculated. The mean genetic distances between groups and the standard errors of the means were calculated using the MEGA v. 6 program by the bootstrap method with 1000 replicates (58). The amino acid distances were evaluated with the Equal Input Model (59).

### Statistical analysis

Data were prepared using GraphPad Prism 9.4.1 (GraphPad Software Inc., San Diego CA, USA) and were expressed as the mean ± standard error of the mean (SEM) or median. Data were analyzed by ANOVA, Wilcoxon matched-pairs signed rank test, or Mann-Whitney to compare three or more groups. Pvalues < 0.05 were used as the threshold for statistical significance. The correlation between neutralization assays was evaluated by Spearman and Pearson correlation analyses.

## Results

### SARS-CoV-2 Spike immunogen design

In previous work we and other have shown that truncation of 13 to 21 amino acids (aa) at the cytoplasmic tail led to efficient incorporation of Spike (S) protein on lentiviral vectors (LV) (40, 60) and that functional mutation of FCS led to incorporation of Spike which was uncleaved at the S1/S2 junction (61, 62). We hypothesized that presence of different Spike conformations on lentiviral particles impacted on ensuing immune response after immunization, similarly to what has been shown with Ad26 vector (63). For immunogenicity studies in the mouse model, we engineered a series of lentiviral transfer vectors (pGAE) (44) expressing different membrane-tethered configurations derived from Wuhan-Hu-1 SARS-CoV-2 Spike protein coding sequence (GenBank: NC_045512.2) (**Table 1** and **Supplementary Figure 1A**), including native full-length wild-type Spike (aa 1-1273, pGAE-Spike), full-length Spike with mutated FCS (RRAR to GSAS) and 2P substitutions (pGAE-S2PF), delta21 wild-type Spike with D614G mutation and 2P substitutions (aa 1-1252, pGAE-S2PGC) and delta21 wild-type Spike with D614G mutation, 2P substitutions and mutated FCS (pGAE-S2PFGC). The Beta (B.1.351) Spike sequence with delta21 and 2P substitutions (pGAE-betaS2PGC) was also generated to assess cross-neutralizing responses compared to the related Wuhan-based vaccine. The schematic representation of the plasmids and the characteristics of the vaccine candidates used in this study are shown in **Table 1** and in **Supplementary Figure 1A**.

**Table 1:** Description of vaccines used in the study.

VACCINE	ANTIGEN	FEATURES
**IDLV-S-wt**	Full-length wild-type SARS-CoV-2 Spike (aa 1-1273, Wuhan-Hu-1 sequence)	Membrane-bound unmodified trimer
**IDLV-S-2PF**	Full-length SARS-CoV-2 Spike with 2P stabilizing aa (K986P, V987P) and mutated furin cleavage site (FCS) (RRAR to GSAS)	Membrane-bound; prefusion S trimer; low S1 shedding
**IDLV-S-2PGC**	SARS-CoV-2 Spike with 2P stabilizing aa, D614G mutation and truncated cytoplasmic tail (CT, delta21 aa)	Higher membrane incorporation; prefusion S trimer; enhanced RBD in up position
**IDLV-S-2PFGC**	SARS-CoV-2 Spike with 2P stabilizing aa, mutated FCS, D614G mutation and truncated CT (delta21 aa)	Higher membrane incorporation; prefusion S trimer; enhanced RBD in up position; low S1 shedding
**IDLV-betaS-2PGC**	SARS-CoV-2 Spike from B.1.351 (beta) VoC with 2P stabilizing aa and truncated CT (delta 21aa)	Higher membrane incorporation; prefusion S trimer; enhanced RBD in up position
**IDLV-HIV_EnvJR_**	HIV JR-FL gp120 (Mock)	Secreted HIV-Envelope monomer
**S-2PFp+Addavax**	SARS-CoV-2 S ectodomain (ECD) with 2P stabilizing aa and mutated FCS (aa 1-1138, Wuhan-Hu-1 sequence)	Prefusion S ectodomain; trimer (subunit vaccine)

Expression of different Spike proteins was evaluated by using flow cytometry on 293T Lenti-X cells transfected with each transfer vectors using a commercial rabbit anti-Spike S2 polyclonal Ab (pAb), three human neutralizing monoclonal Abs (mAbs) (COVA2-15, COVA1-16 and COVA1-18) that recognize different epitopes of RBD (47), one non-RBD-binding neutralizing mAb (COVA1-21) and the SARS-CoV-1 neutralizing mAb CR3022 (64), which recognizes a conserved weakly neutralizing cryptic epitope in SARS-CoV-2 Spike, accessible in the open conformation of the protein with at least 2 RBD in the up-conformation, not overlapping with the ACE2 binding site within SARS-CoV-2 RBD (65–67). All membrane-bound Spikes were recognized by anti-S2 antibody, indicating their correct expression on the membrane of transfected cells (**Supplementary Figure 2**). The human mAbs COVA2-15, COVA1-16, COVA1-18 and COVA1-21 recognized all Wuhan-Hu-1 derived Spikes, whereas the beta variant was not recognized by COVA1-18 mAb, and poorly recognized by COVA2-15 and COVA1-21 mAbs. Interestingly, mAb CR3022 was overtly reactive only against Spike with mutated FCS, suggesting that this mAb recognizes an epitope that is accessible only in this Spike configuration.

### IDLV-CoV2 are decorated with membrane tethered Spike proteins

**Figure 1:**
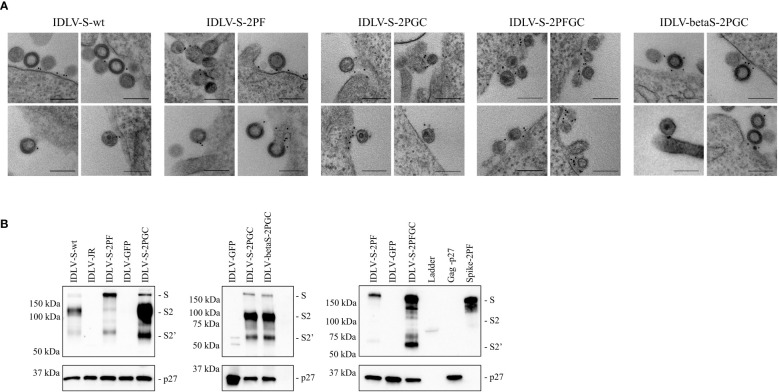
**(A)** IDLVs are pseudotyped with membrane-tethered Spike proteins. 293T Lenti-X cells producing the different IDLV-CoV2 were probed with anti-RBD COVA2-15 or COVA1-16 human mAbs and observed by TEM. Four representative images are shown for each sample. Bars, 0.2 μm. Shown are results from one representative of n = 2 experiments. **(B)** Spike incorporation on IDLV-CoV2 particles used for immunization. Western blot of lysates from concentrated preparation of IDLV-CoV2 pseudotyped with the different Spike conformations. IDLV-GFP and IDLV-JR are included as mock controls and SIV-Gag p27 protein (50 ng) as positive control. Filters were probed with polyclonal anti-S2 and anti-p27 Gag Abs. Molecular weights are indicated on the left. Bands corresponding to S, S2 and S2’ representing full-length and S2 or S2’ portion, are indicated. Blots derive from the same experiment and were processed in parallel. Shown are results from one representative of n = 3 experiments.

**Figure 2:**
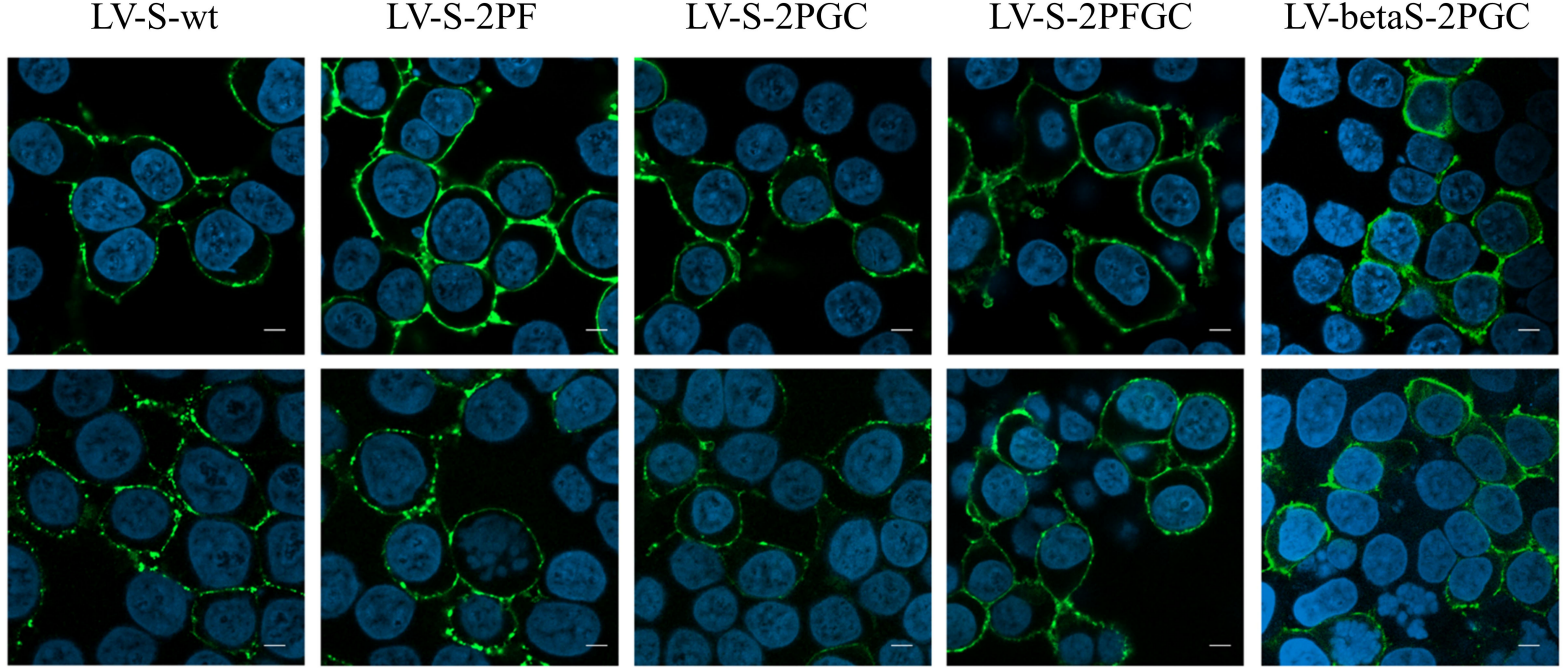
Spike expression from lentiviral vectors. Analysis by CLSM of 293T Lenti-X cells transduced with the indicated lentiviral vectors expressing the different Spike conformations. Cells were stained with anti-RBD COVA2-15 or COVA1-16 human mAbs followed by anti-human IgG Alexa Fluor 488 as secondary Ab (green). Nuclei were stained in blue with DAPI. Two images representing single central optical sections are shown for each sample. Shown are results from one representative of n = 3 experiments. Scale bar is 10 μm.

IDLV expressing the different conformations of Spike (IDLV-CoV2) were produced to evaluate incorporation on pseudovirions. Transmission electron microscopy (TEM) images of 293T Lenti-X cells producing the IDLV-CoV2 showed presence of S on the released IDLVs and plasma membranes of the producing cells after staining with COVA2-15 or COVA1-16 mAbs, the latter used to stain cells producing IDLV-betaS-2PGC (**Figure 1A**). To further assess incorporation of the different configurations of Spike on pseudovirus, recovered and concentrated preparations of IDLV-CoV2 to be used for immunization were normalized for amount of p27 Gag and analyzed by Western blot (WB) using anti-S2 (S2 fragment) and anti-p27 Gag polyclonal antibodies (**Figure 1B**). Gag protein was detected similarly in all IDLV preparations and bands corresponding to the Spike protein were detected in all IDLV-CoV2 concentrated preparations, except for IDLV-GFP and IDLV-JR, encoding the codon optimized HIV-1_JR-FL_ gp120 (32), as expected. In particular, the highest amount of Spike was incorporated in the IDLVs delivering the delta21 C-truncated version of the protein (IDLV-S-2PGC, IDLV-betaS-2PGC and IDLV-S-2PFGC), compared to IDLV-S-wt and IDLV-S-2PF. Since the removal of the FCS results in less S1 shedding of the S protein, IDLV-S-2PF and IDLV-S-2PFGC showed mainly presence of the stabilized uncleaved full-length S protein, while in both IDLV-S-2PGC (Wuhan-Hu-1 and Beta) and IDLV-S-wt the majority of the protein was cleaved at the FCS and showing on WB the S2 portion with the anti-S2 polyclonal Ab (**Figure 1B**). Interestingly, the presence of S2’ fragment in IDLV-CoV2 preparations incorporating high levels of S might be due to Spike engagement of low levels ACE2 expression on 293T cells (**Supplementary Figure 3** **)** during IDLV production, which primes S2′ site cleavage (68). These results demonstrate that different configurations of Spike are incorporated differently and according to their design in the vector particles used to immunize mice. To appreciate the membrane location of Spike expressed by the vector, 293T Lenti-X cells were transduced with LV pseudotyped with VSV.G protein and delivering the different Spikes, stained with anti-RBD human COVA2-15 or COVA1-16 mAbs and examined by confocal microscopy (CLSM) (**Figure 2**). The membrane expression of Spike was evident in the cells transduced with any vector, confirming their ability to express membrane bound antigens.

### A single immunization of IDLV-CoV2 elicits strong and persistent antibody responses

**Figure 3:**
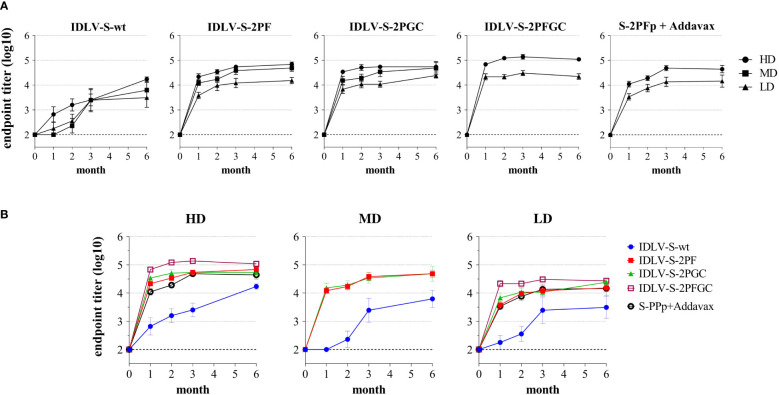
Kinetics of anti-RBD binding antibodies in immunized mice. BALB/c mice (N=5-6 animals per group) were vaccinated once with escalating doses (High dose, HD: 25x10^6^; Medium dose, MD: 6.25x10^6^; Low Dose, LD: 1.56x10^6^ RT units/mouse) of SIV-based IDLVs expressing Spike or HD and LD of the purified Spike protein (10 and 1 µg/mouse) adjuvanted with Addavax. Anti-RBD IgG antibodies were evaluated at different time points (month) from immunization in sera of mice immunized with the indicated vaccines. Results are expressed as log10 mean endpoint titer ± SEM. The dotted line indicates the assay cut off (minimum serum dilution tested 1:100). **(A)** Each graph shows the results from mice of the same vaccine group at HD, MD and LD. **(B)** Each graph shows different vaccine groups at the same dose.

To evaluate the magnitude, quality and persistence of anti-Spike immunity, BALB/c mice were injected once intramuscularly (i.m.) with escalating doses of IDLV-CoV2 vaccines (1.56x10^6^, 6.25x10^6^ and 25x10^6^ RT Units/mouse, hereafter indicated as Low Dose-LD, Medium Dose-MD and High Dose-HD) and compared to mice immunized with a subunit vaccine based on the purified S-2PF trimeric protein, at two different doses (10 and 1 µg/mouse) together with Addavax as an adjuvant. The kinetics of anti-RBD IgG titers in serum was evaluated by ELISA at 0, 1, 2, 3 and 6 months after immunization, using the Wuhan RBD monomer as coating protein (45). The kinetics of serum Ab response of each group and at each dose are depicted in **Figures 3A, B**. All vaccine candidates induced anti-RBD IgG Abs, according to the injected dose, starting from 1 month (the first point post-immunization evaluated) and increasing over time, mostly reaching a plateau at 3 months. All groups maintained the response up to 24 weeks, the experimental endpoint, at different levels. Overall, IDLV-S-wt induced the lowest and IDLV-S-2PFGC the highest anti-RBD Ab response at all injected doses (ANOVA, p<0.05). IDLV-S-2PF, IDLV-S-2PGC and the subunit vaccine induced similar Ab responses (ANOVA, p>0.05). IDLV-S-wt showed a peculiar kinetics of Ab response, increasing over time up to 6 months, reaching levels of Abs similar to the other vaccine candidates (**Figures 3A, B** **)**.

### Level, kinetics and cross-neutralization of neutralizing antibodies are dependent on the immunogen configuration

Analysis of neutralizing Abs (nAbs) in serum of immunized mice was performed utilizing the pseudovirus assay, which is based on LV expressing luciferase (LV-Luc) pseudotyped with the truncated form of Spike derived from Wuhan-Hu-1 and VoC, as we previously described (40, 45, 46). A virus neutralization (VN) assay using the original infectious SARS-CoV-2 (isolate SARS-CoV-2/human/ITA/INMI1/2020) was initially performed using 31 serum samples to compare the assays and correlate the titers (ID50). The neutralization titers calculated using the two different assays correlated well with each other, as indicated by Spearmen and Pearson r values (**Supplementary Figure 4**). We observed a statistically significant difference between ID50 values, being the ID50 calculated using the pseudovirus neutralization assay higher than ID50 derived from infectious virus based test (median ID50: 1589 and 1280 in pseudovirus and infectious virus assays, respectively) probably due to higher sensitivity of the Luc-based assay.

**Figure 4:**
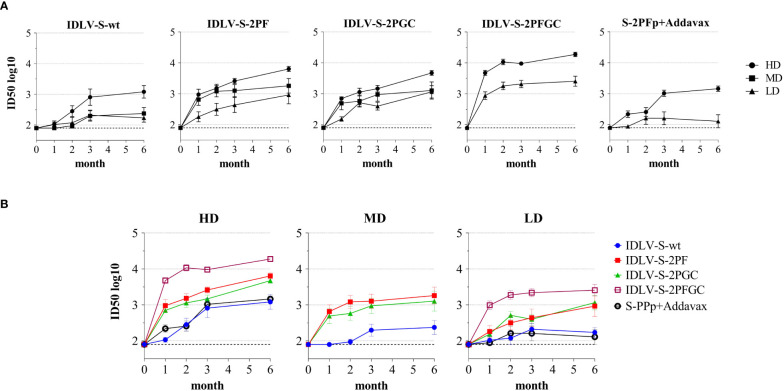
Kinetics of neutralizing antibodies in immunized mice. Anti-Wuhan neutralizing antibodies (nAbs) were evaluated at different time points (month) from immunization in sera of mice immunized with the indicated vaccines. Results are expressed as mean ID50 ± SEM with N=5-6 animals per group. HD, MD and LD: high, medium and low dose of vaccine, respectively. The dotted line indicates the assay cut off (minimum serum dilution tested 1:80 dilution). **(A)** Each graph shows results from mice of the same vaccine group at HD, MD and LD. **(B)** Each graph shows different vaccine groups at the same dose.

The pseudovirus-based assay was used for the evaluation of presence and magnitude of nAbs over time in all immunized mice (**Figure 4**). All IDLV-CoV2 vaccines induced different levels of nAbs according to the injected dose within the same vaccine formulation (**Figure 4A**). Interestingly, the level of nAbs increased over time up to 6 months, especially in the HD treated animals, including mice immunized with the subunit vaccine at HD. Overall, IDLV-S-2PFGC induced the highest nAb response, while IDLV-S-wt induced the lowest response compared to all the other IDLV groups and at any dose, particularly evident at early time points (**Figure 4B**), thus in part paralleling the results obtained after evaluation of the binding Abs (**Figure 3**), but with a more evident difference among groups. In particular, IDLV-S-2PF and IDLV-S-2PGC consistently induced a higher and more rapid nAb response compared to that in mice immunized with S-2PFp+Addavax (ANOVA, p<0.05). The nAb response was barely detectable at 6 months in the sera of mice immunized with the subunit vaccine at LD.

IDLV-betaS-2PGC vaccine, expressing the Beta Spike, was evaluated in the immunization protocol in order to assess and compare the cross-neutralizing responses using a Spike from the VoC able to better escape from the Wuhan-Hu-1 S vaccine induced nAbs, at the time when this study was conducted (69). A comparison of the anti-Wuhan Ab and nAb responses after immunization with IDLV-S-2PGC and the corresponding Beta variant IDLV-betaS-2PGC is shown in **Supplementary Figure 5**. The levels of both binding and nAbs were very similar in both vaccine groups at any dose and time point tested (Wilcoxon matched-pairs signed rank test, p>0.05).

**Figure 5:**
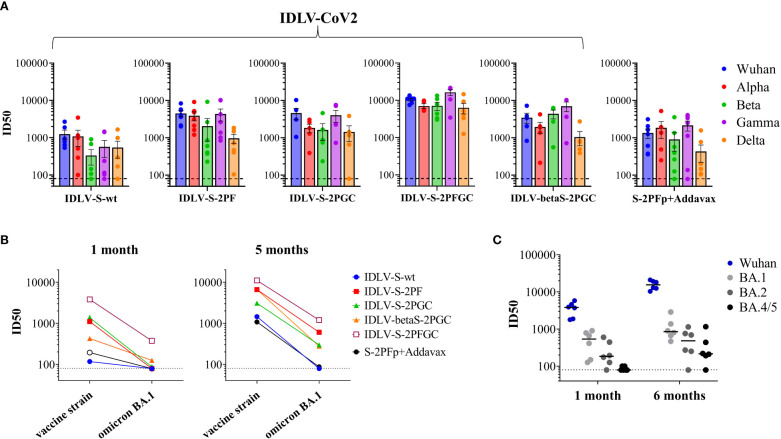
Cross-neutralization with VoC. **(A)** NAbs against each VoC were measured in serum samples collected at 4-5 months after immunization with HD vaccines, using LV-Luc pseudotyped with Spike from the indicated VoCs. Results are expressed as mean ID50 ± SEM. Each dot represents a single mouse. **(B)** Serum samples from early and late time points after immunization with HD vaccines were analyzed for cross-neutralization against the Omicron BA.1 variant. Results are expressed as median ID50. **(C)** Sera from IDLV-S-2PFGC were assayed for cross-neutralization against Omicron VoC at early and late time points after vaccination. Each dot represents a single mouse. The black line indicates the median ID50. The dotted lines indicate the assay cut off (minimum serum dilution tested 1:80 dilution).

Serum samples from all IDLV-CoV2 HD vaccinated mice, including IDLV-betaS-2PGC, were assayed for cross-neutralization ability using pseudoviruses pseudotyped with Spike from Alpha, Beta, Gamma, Delta and Omicron BA.1 VoC. Due to the small amount of mouse serum collected at each time point, 4 and 5 month-sera were dedicated to this analysis. As shown in **Figure 5**, the pattern of cross-neutralization is similar to that observed in human subjects vaccinated with Wuhan-based vaccines (46, 70, 71). Overall, compared to the Wuhan strain, homologous to the vaccine sequence, ID50 values of Alpha were >Gamma>Delta>Beta>Omicron. However, pattern and magnitude of neutralizing responses were different according to the Spike configuration delivered by each IDLV. In particular, we observed a high fold reduction of the ID50 against Delta compared to the vaccine strain in all vaccinated mice (**Table 2**), except for the IDLV-S-2PFGC group (2.41 fold reduction), where the vaccine induced nAbs were efficiently able to block the Delta pseudoviruses on target cells (**Figure 5A** and **Table 2**). Overall, IDLV-2PFGC, combining all modifications, outperformed the other candidates in terms of nAbs magnitude at early and later time points and breadth of neutralization against all VoC, including the Omicron BA.1 variant, although the latter at lower levels (**Figure 5B**). The IDLV-betaS-2PGC delivering the Beta Spike induced a better neutralization against Beta and Gamma pseudoviruses compared to the corresponding parental Wuhan-Hu-1 S immunogen (IDLV-S-2PGC), but neutralization against the Omicron BA.1 VoC was substantially reduced (**Figure 5** and **Table 2**). Sera from IDLV-S-2PFGC immunized mice were also evaluated for neutralization of pseudoviruses enveloped with Spike protein from additional circulating Omicron BA.2 and BA.4/5 variants. As shown in **Figure 5C**, both at 4 and 24 weeks after immunization, BA.2 and BA.4/5 pseudoviruses were neutralized to levels lower than the parental BA.1.

**Table 2:** Median ID50 fold reduction compared to the vaccine strain.

	WT	Alpha	Beta	Gamma	Delta	Omicron
**IDLV-S-wt**	1.00	1.87	13.27	3.67	3.91	20.00
**IDLV-S-2PF**	1.00	1.14	5.78	1.56	5.06	6.00
**IDLV-S-2PGC**	1.00	2.34	3.50	1.36	5.17	5.69
**IDLV-S-2PFGC**	1.00	1.46	1.96	0.59	2.41	9.09
**S-2PFp+adj**	1.00	1.25	2.85	0.61	3.43	2.00
**IDLV-betaS-2PGC**	1.33	2.60	1.00	0.62	3.34	18.00

### Th-type T cell immunity persists up to six months from the immunization in IDLV-immunized mice

**Figure 6:**
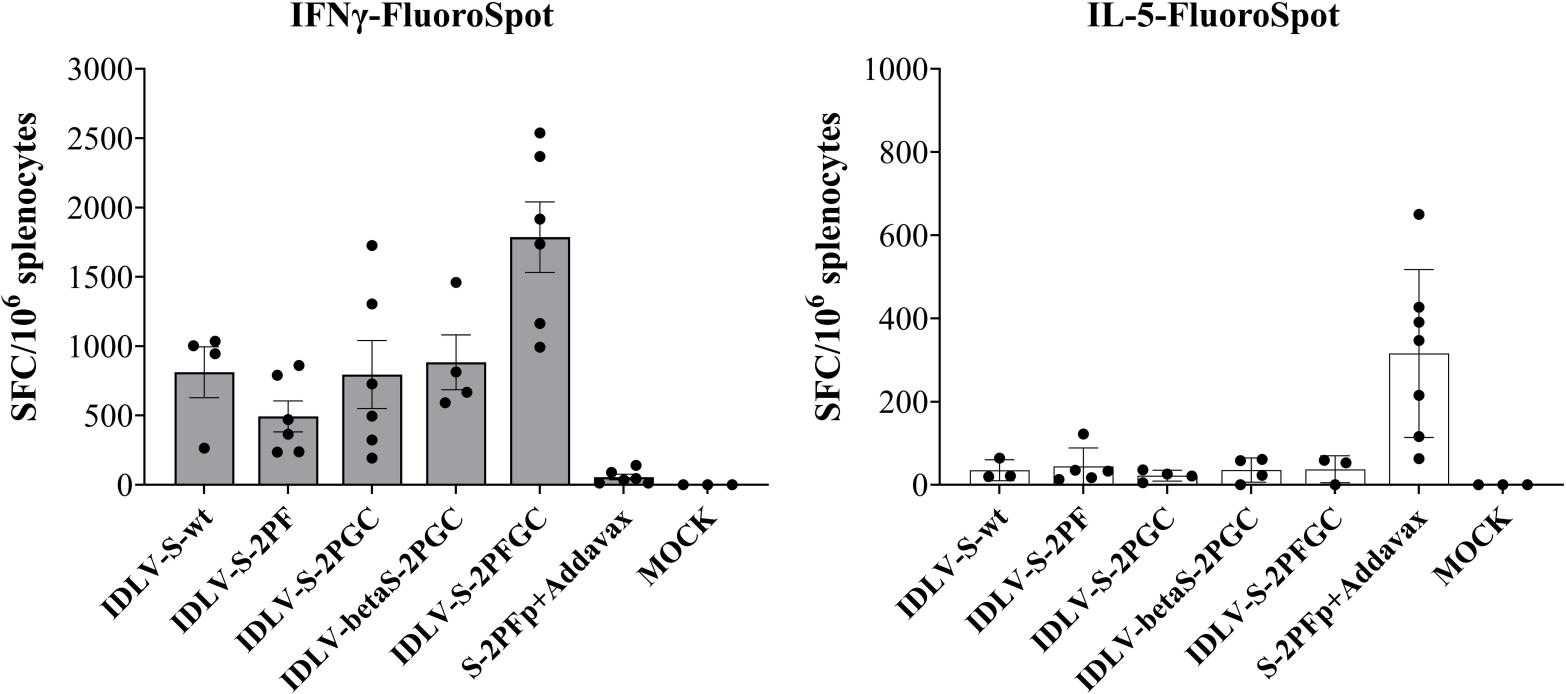
T cell immunity persists for six months after a single immunization. Splenocytes from HD groups recovered at 6 months after immunization were assayed for IFNγ (left panel) and IL-5 (right panel) producing T cells by IFNγ/IL-5-FluoroSpot. Cells were stimulated over night with Wuhan-Spike peptide pool. Data are expressed as mean ( ± SEM) specific Spot Forming Cells (SFC) per million splenocytes. Each dot represents a single mouse.

Since the S-specific T cell response may contribute to the vaccine efficacy, at least in terms of control of the clinical manifestation, IFNγ/IL-5 FluoroSpot was performed on splenocytes at 6 months after immunization in the HD groups using pools of peptides covering the entire Wuhan-Spike protein. As shown in **Figure 6**, Spike-specific IFNγ producing T cells were induced in all mice vaccinated with any IDLV, with IDLV-S-2PFGC showing the highest response compared to the other groups (Mann-Withney test p<0.001 vs S-2PF protein immunized animals; p<0.05 vs all other groups). Mice immunized with the protein subunit vaccine showed instead IL-5-producing T cells, as expected, since Addavax is a Th2-type adjuvant, which favours the induction of IL-5 (**Figure 6**) (72). Conversely, none of IDLV-vaccinated mice showed IL-5-producing T cells when stimulated with the Spike peptide pool, confirming the Th1-type profile of T cells induced by IDLV vaccination.

### Genetic distance among Wuhan and Omicron helps explain the escape from nAb responses

**Figure 7:**
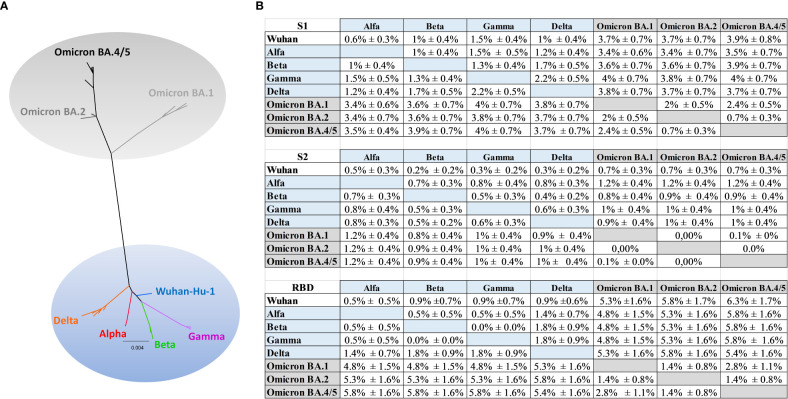
Phylogenetic analysis. **(A)** Maximum likelihood phylogenetic tree inferred from Spike amino acid sequences. The ancestral SARS-COV-2 sequence (Wuhan/Hu- 1/2019), the Alpha, Beta, Gamma, Delta and the Omicron (BA.1, BA.2, BA.4/5) variants are highlighted with different colors. The scale bar at the bottom of the tree corresponds to amino acid substitutions per site. The authors, originating and submitting laboratories of the sequences from GISAID used to generate the figures are listed in **Supplementary Table 1**. **(B)** Mean distances ± SEM at amino acid level for S1, S2 and RBD.

In order to better understand the limited antibody cross-reactivity to Omicron in sera of mice immunized with the optimized Wuhan-derived Spike, a phylogenetic analysis using amino acid sequences for the full-length Spike, RBD, S1 and S2 portions was performed. The maximum likelihood (ML) phylogenetic trees for the Spike, S1, S2 and RBD were shown in **Figure 7A** and **Supplementary Figure 6**. The ML tree performed on the Spike (**Figure 7A**) showed the SARS-CoV-2 variants located into two main supported clades. At one end of the tree the Wuhan strain and the Alpha, Beta, Gamma and Delta VoC were identified. On the other end, a supported clade including the Omicron variants (Omicron BA.1, BA.2, BA.4/5) was shown. In particular, the Omicron BA.2 appeared related and closer to BA.4/5 (**Figure 7A**), in respect to BA.1.

The phylogenetic analysis (**Supplementary Figure 6**) showed a higher distance (**Figure 7B**) between the group including Wuhan, Alpha, Beta, Gamma, Delta and the group including the Omicron (BA.1, BA.2, BA.4/5) in S1 respect to S2 portion, and a higher distance in RBD respect to S2 or to S1 (**Figure 7B**). In particular, the mean diversity at amino acid level between Wuhan and Omicron BA.1, BA.2, BA4/5 increased from 0.7% ± 0.3% (distance ± standard error) for S2 alignment to 3.7% ± 0.7% (Omicron BA.1, BA.2) and to 3.9% ± 0.8% (Omicron BA4/5) in the S1 alignment **(** **Figure 7B** **)**, meanwhile increased to 5.3% ± 1.6% (Omicron BA.1) to 5.8% ± 1.7% (Omicron BA.2) and to 6.3 ± 1.7% (Omicron BA4/5) in RBD. Similarly, the mean genetic distance between Beta and Omicron increased from 0.8% ± 0.4%/0.9% ± 0.4% in S2 alignment to 3.6% ± 0.7% (Omicron BA.1, BA.2) and to 3.9% ± 0.7% (Omicron BA4/5) in the S1 alignment **(** **Figure 7B**); meanwhile the mean distance in RBD increased to 4.8% ± 1.5%, 5.3% ± 1.6%, 5.8% ± 1.6% respectively for Beta and Omicron BA.1, Beta and BA.2, Beta and BA.4/5.

## Discussion

In this study we exploited SIV-based IDLVs delivering different configurations of SARS-CoV-2 Spike in order to induce a long-term, high-magnitude and cross-lineage neutralizing Ab response. We hypothesized that a strategy combining a rationally optimized trimeric Spike on the vector particles together with the sustained immunity provided by IDLV could enhance the quality of the humoral response after a single immunization.

In previous works, we and others showed that membrane-tethering of HIV-1 Env on the surface of a virus-like particles (VLP) or IDLV elicited broader immune responses compared to soluble HIV-Env (34, 73) and that lentiviral vectors can be successfully pseudotyped with SARS-CoV-2 Spike glycoprotein (40). Here, we show that IDLV-CoV2 can be pseudotyped with different configurations of membrane-tethered Spike, and that Spike protein design strongly affects incorporation on vector particles and the resulting immune responses elicited by IDLV. In particular, we evaluated the incorporation of different forms of Spike protein on lentiviral particles and investigated the importance of the immunogen design in the context of the IDLV, an enveloped viral vector which we have started exploiting for its ability to be pseudotyped with membrane-tethered glycoproteins. We reasoned that modifications aimed at increasing incorporation of full-length Spike on IDLV particles would have improved the immune response induced by immunization with IDLV expressing rationally designed immunogens. Our results confirmed that truncation of 21 aa at the C-terminus of the cytoplasmic tail greatly enhanced membrane incorporation of Spike on viral particles, as also observed in other settings (40, 69, 74, 75). Importantly, replacement of RRAR with GSAS at the FCS prevented S1/S2 cleavage, leading to incorporation of a higher amount of full-length Spike protein on IDLV. We hypothesized that this feature may favour the elicitation of nAbs compared to IDLV expressing Spike with an intact FCS, due to the higher amount of S1-associated neutralizing epitopes present on IDLV particles that can be sensed immediately by immune cells after the immunization. To further ameliorate the quality of the anti-Spike humoral response, we evaluated mutations aimed at improving exposure of neutralizing epitopes, including the introduction of 2P substitutions (K986P and V987P) for stabilizing Spike in the prefusion conformation (76, 77) and the inclusion of the D614G mutation, for enhancing RBM exposure (42) and Spike protein density (78) in the IDLV. These modifications have been already adopted, alone or in combination, using different Spike delivery systems, including adenoviral vectors (63, 79, 80), MVA (81) and mRNA vaccines (82, 83).

To assess and compare the immunogenicity of IDLV delivering different configurations of membrane-tethered Spike, mice were immunized once i.m. with scalar amounts of different IDLV-CoV2. Data clearly indicated that the prefusion stabilized Spike with 2P substitutions and mutated FCS, preventing S1/S2 cleavage, was a more effective immunogen in terms of level and persistence of autologous Ab and nAb response compared to the wild-type Spike which showed the poorest humoral immunity (IDLV-S-wt vs IDLV-S-2PF), in line with data from other studies (81, 84). We also investigated the role of the delivery system in the induction of humoral response, comparing IDLV delivering the purified prefusion stabilized Spike with 2P substitutions and mutated FCS (IDLV-S-2PF) with the same version of the Spike delivered as subunit vaccine administered with a squalene-based adjuvant. The level and quality of nAb responses following immunization with IDLV were significantly higher at any dose and time point analyzed.

Immunization with IDLV expressing membrane-tethered D614G Spike with truncated cytoplasmic tail induced higher IgG and autologous nAb levels compared to IDLV expressing full-length Spike (IDLV-S-wt vs IDLV-S-2PGC and IDLV-S-2PF vs IDLV-S-2PFGC), which was more evident at the early and late time points and with any dose of the injected IDLVs. Overall, IDLV-2PFGC, combining all the Spike modifications, outperformed the other candidates, in terms of level of both binding Abs and nAbs at early and later time points, showing the best ability to neutralize all tested VoC, including the circulating Omicron variants, although to a lesser extent. The high-density exposure of the full-length, stabilized and cytoplasmic tail truncated Spike trimers on the IDLV particles, avoiding S1 shedding, was likely responsible for the optimal induced immunity. Interestingly, the pattern of cross-neutralization was different among the tested Spike delivered by IDLVs, underlying the key role of the immunogen in driving the induction of functional nAbs. The Beta Spike variant delivered by IDLV (IDLV-betaS-2PGC) induced a better neutralization against autologous Beta and heterologous Gamma variants compared to the corresponding parental Wuhan-Hu-1 Spike immunogen (IDLV-S-2PGC), but did not improve the cross-neutralization of the other VoC, especially Omicron BA.1. Interestingly, recent data showed that the use of a vaccine adapted to the Omicron VoC may be beneficial to counteract the homologous variant (85), but can be detrimental for blocking the other known variants and potentially also some future new variants (86). This further underlines the importance of iterative testing and optimization of the antigen/vector combination to be included in the vaccine formulation. Indeed, in this study, immunization with IDLV-S-2PFGC, delivering fully optimized Wuhan Spike, led to high level of vaccine-induced immunity to SARS-CoV-2 against all early VoC including Alpha, Beta, Gamma and Delta. On the other hand, the same sera exhibited increased resistance to neutralization against Omicron VoC (BA.1>BA.2>BA.4/5). Evasion of immunity by the Omicron VoC may be attributable to the high number of amino acid substitutions which are present mostly in the S1 subunit of the Spike protein, as indicated by the higher genetic distance between Omicron and all the other VoC. Given the higher number of mutations in Spike, Omicron subvariants may be considered as belonging to a distinct SARS-CoV-2 group or serotype, as recently suggested (87). In addition to the number of mutations, also the effect on the conformation, structure and the combination of the effect of several mutations need to be considered. In this context, previous works have shown that the numerous mutations observed in the Omicron variants alter the conformation of antigenic sites (88, 89), leading to immunological escapes from neutralizing antibodies induced by the Wuhan-based vaccine. The procedure we followed for improving immunity against Spike may be successful against variants within a viral strain that contains a limited number of mutations, whereas a vaccine active against highly divergent strains should focus on portions of Spike that are more conserved, such as the S2 portion (90) where the genetic distance among VoC is much lower. Also, the vaccine should include more conserved viral proteins that induce effector CD8 responses in addition to humoral responses (91). Finally, since intranasal immunization with a different IDLV delivering the ancestral full-length unmodified Spike in ACE2+ rodent animal models showed evidence of protection after challenge (37, 38), a mucosal vaccine based on the optimized Spike delivered by IDLV may be the key for the induction of persistent and cross-neutralizing responses at the portal entry of the virus.

In this study, after a single immunization in mice, nAbs increased over time, suggesting a maturation of B cell response allowing for the maintenance of B cell memory and the development of long lived plasmacells. This phenomenon is not observed in vaccinated humans, where nAb response wanes over time (46, 92) and suggests that some immunological aspects, including analysis of persistence of immunity and breadth of immune responses, should be verified in other animal models, such as non-human primates (93). Interestingly, a recent report showed that after three doses of mRNA vaccine in humans, the Spike-specific repertoire of human B cells was significantly increased, allowing for the development of high affinity and cross-neutralizing antibodies (94), suggesting that multiple antigen exposure is essential for B cell maturation. A recent report elegantly demonstrated that a slow-delivery (12 days) immunization approach and a long-prime in the non-human primate immunogenicity model induced a remarkable germinal center duration and B cell maturation resulting in an enhanced quality of antibody response (95). In this context, we previously demonstrated the persistence of antigen expression at the site of immunization and in draining lymph nodes after IDLV immunization (96) and the durability of IDLV-induced immunity in mice using IDLV delivering different viral antigens, including HBV-HBsAg (97), Influenza HA (39) and a membrane tethered HIV-1 Envelope sequence, the latest confirmed also in non-human primates (34). Persistent production of Spike protein upon immunization with IDLV may favour triggering of maturation of immune response, eventually resulting in higher antibody potency and breadth.

In addition to the humoral response, the T cell immunity represents a key element of the adaptive immune response to SARS-CoV-2 that contributes to reduce the virus replication and eventually the disease progression (91, 98). Previous work has shown that most of the SARS-CoV-2 T cell epitopes defined by different studies (99, 100) are conserved within VoC and consistently with this observation, the antigens containing the sequence variations are cross-recognized by infected and/or vaccinated individuals (101). Due to the high number of different epitopes and the epitope breadth on an individual level, the T cell escape appears unlikely. Those observations underline the importance of inducing T cell immunity following vaccination. In this study, Spike-specific IFNγ producing T cells were detected in all IDLV vaccinated mice at 6 months after immunization, confirming the ability of IDLV to elicit effective and durable T cell immunity (102).

The induction of long-term immunity provided by IDLV combined with rationally designed immunogens delivered both as transgene and pseudotyping glycoprotein on the lentiviral particles, resulted in a versatile and efficient platform to be exploited as a successful vaccine candidate against SARS-CoV-2. To confirm and validate these promising results, IDLV-S-2PFGC will be used in the non-human primate model of immunogenicity and infection, compared with the mRNA vaccines.

This study has some limitations. While IDLV pseudotyping and cross-lineage S-specific nAbs were demonstrated, the study was conducted using a small-animal model, without challenging with live virus. Although presence of nAbs correlates with protection (103), it is yet unknown the minimum level of nAbs which are required for providing protection after the infection, particularly in the mucosa of the respiratory tract. In this context, a study using an HIV-based IDLV expressing full-length wild type Wuhan-Hu-1 Spike in a prime-boost regimen in hamsters, showed strong reduction of viral copies after challenge with homologous SARS-CoV-2 (37), suggesting that an optimized Spike delivered by IDLV will be a promising vaccine strategy.

## Data availability statement

The original contributions presented in the study are included in the article/**Supplementary Material**. Further inquiries can be directed to the corresponding authors.

## Ethics statement

The animal study was authorized by the Italian Ministry of Healthy and reviewed by the Service for Animal Welfare at Istituto Superiore di Sanità (Authorization n. 731/2020-PR, 21/7/20, prot. D9997.107).

## Author contributions

DN and ACara conceived and designed the study, analyzed the data, and wrote the manuscript. MB supervised animal experiments, performed ELISA and performed T-cell response experiments. AG and MP prepared the plasmids, the lentiviral vectors, performed flow cytometry experiments and neutralizing antibody assays. MLDA performed neutralizing antibody assays. ACan performed the Western blot assays. ZM performed ELISA and VN assay. SC performed confocal microscopy experiments. AT performed electron microscopy experiments. CF prepared plasmids. SM, ACap, MC, AI and RN produced the recombinant RBD protein. AV performed the animal experiments. MG and RS provided reagents and helped in study implementation. AP conceived and performed phylogenetic analyses. All authors contributed to the article and approved the submitted version.
