# Supplementary material for: Different configurations of SARS-CoV-2 spike protein delivered by integrase-defective lentiviral vectors induce persistent functional immune responses, characterized by distinct immunogenicity profiles
Source: Front Immunol. 2023 Apr 5;14:1147953. doi: 10.3389/fimmu.2023.1147953 (PMC10113491; doi:10.3389/fimmu.2023.1147953)
Supplement: Supplementary file 1 [file DataSheet_1.pdf]

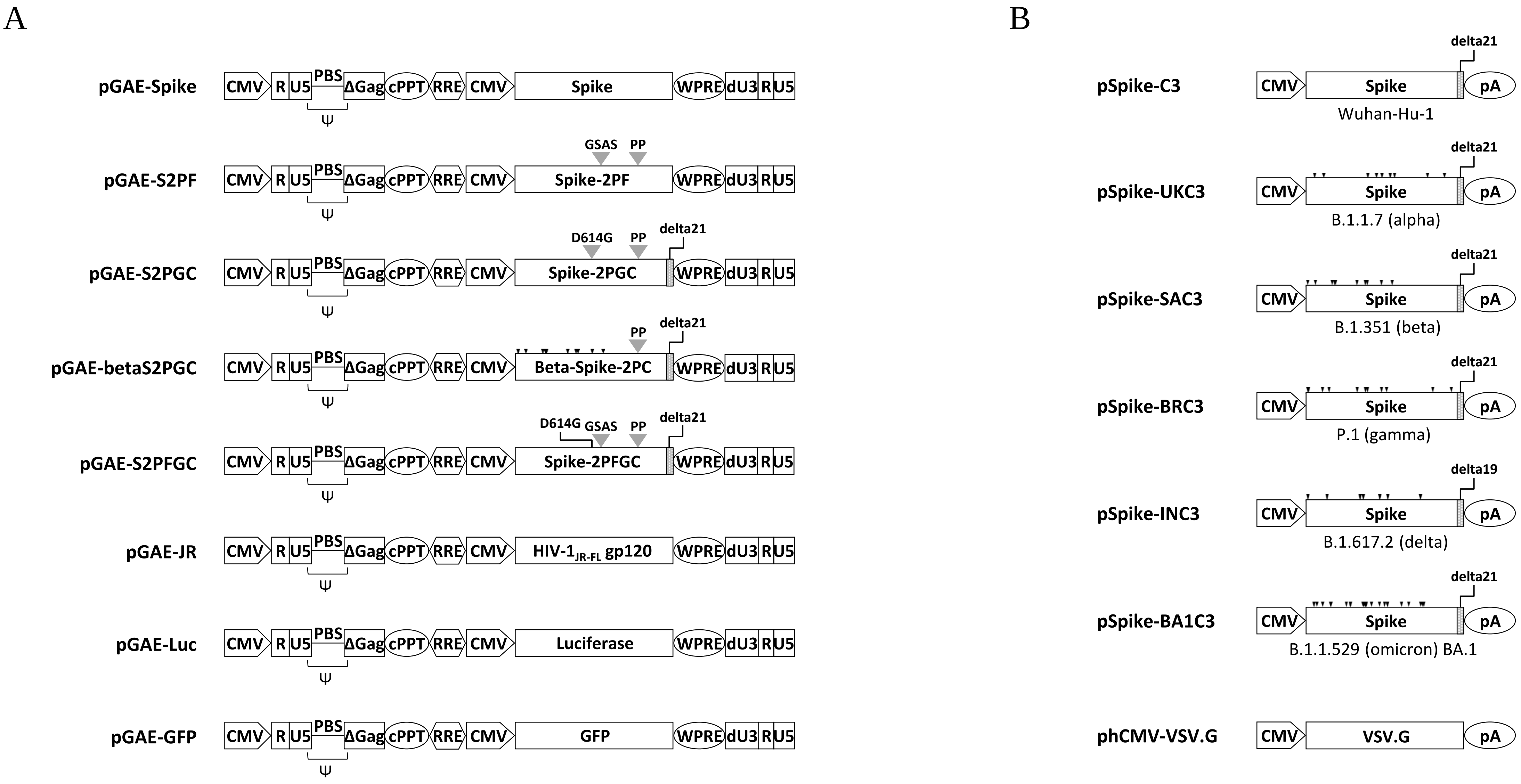

Supplementary Figure 1. **Schematic representation of transfer vectors and plasmids used in this study**. (**A**) Lentiviral transfer vectors expressing Spike proteins, HIV-1$_{JR\text{-}FL}$ gp120, Luciferase and GFP. (**B**) Pseudotyping plasmids expressing cytoplasmic tail truncated wild-type Spike (pSpike-C3), VoC (pSpike-UKC3, pSpike-SAC3, pSpike-BRC3, pSpike-INC3, pSpike-BA1C3) and VSV.G envelope (phCMV-VSV.G). CMV, cytomegalovirus immediate-early promoter; R, repeat element; U5, 5' untranslated region; U3, 3' untranslated region; PBS, primer binding site; Ψ, packaging signal; cPPT, central polypurine tract; RRE, Rev response element; dU3, SIN deletion in U3 region of 3' LTR; WPRE, woodchuck hepatitis virus post-transcriptional regulatory element. See Methods for details on construction.

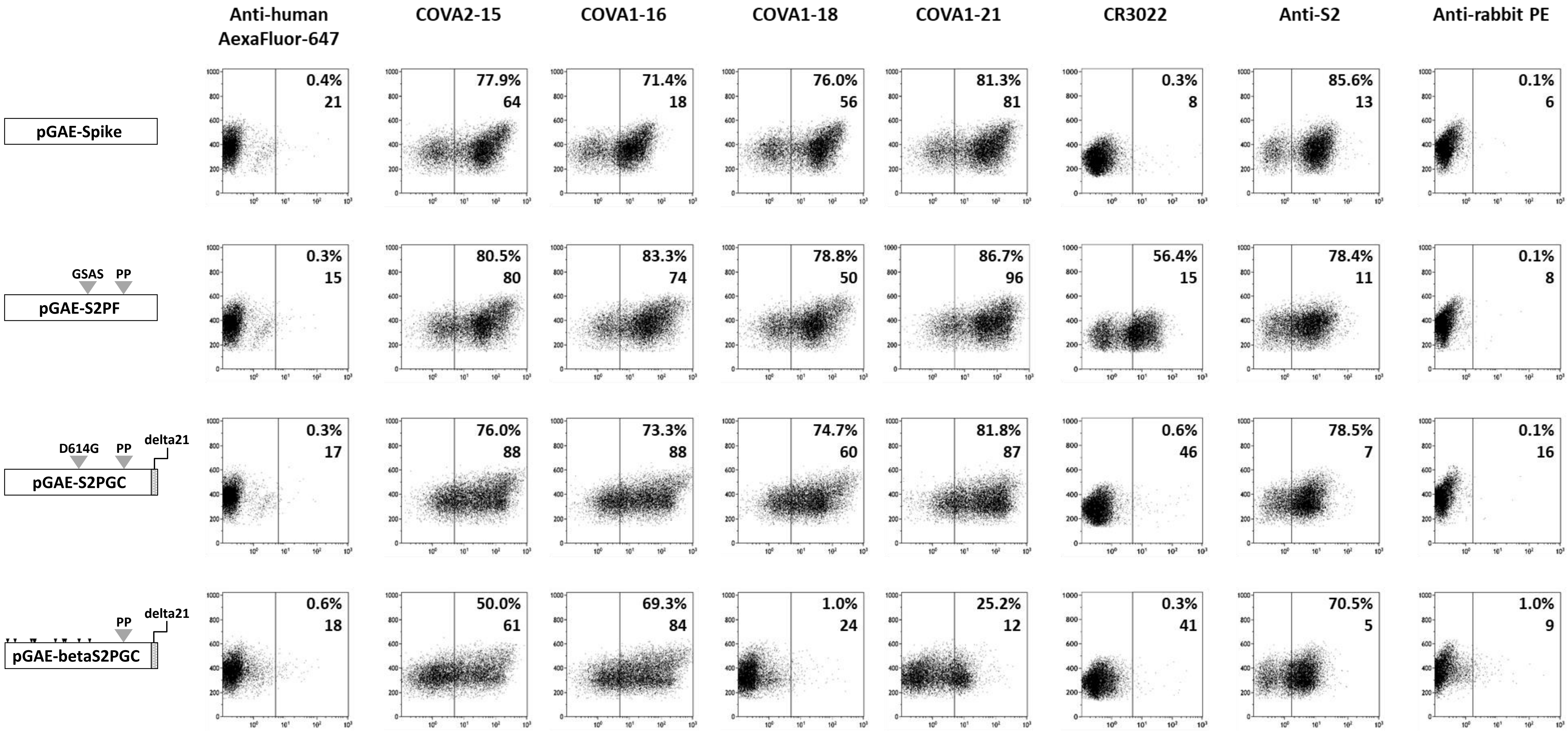

Supplementary Figure 2. **Spike expression in 293T Lenti-X transfected with Spike-expressing lentiviral transfer vector plasmids.** Cells transfected with the indicated pSpike plasmids were stained with anti-RBD neutralizing mAbs (COVA2-15, COVA1-16 and COVA1-18), one non-RBD-binding neutralzing mAb (COVA1-21), the SARS-CoV-1 neutralizing mAb CR3022, a commercial anti-S2 polyclonal antibody and secondary antibodies alone Anti-human AexaFluor-647 and Anti-rabbit PE). The number within the histogram plot indicates the % of positive cells and the mean fluorescence intensity (MFI). Shown are results from one representative of n = 3 experiments.

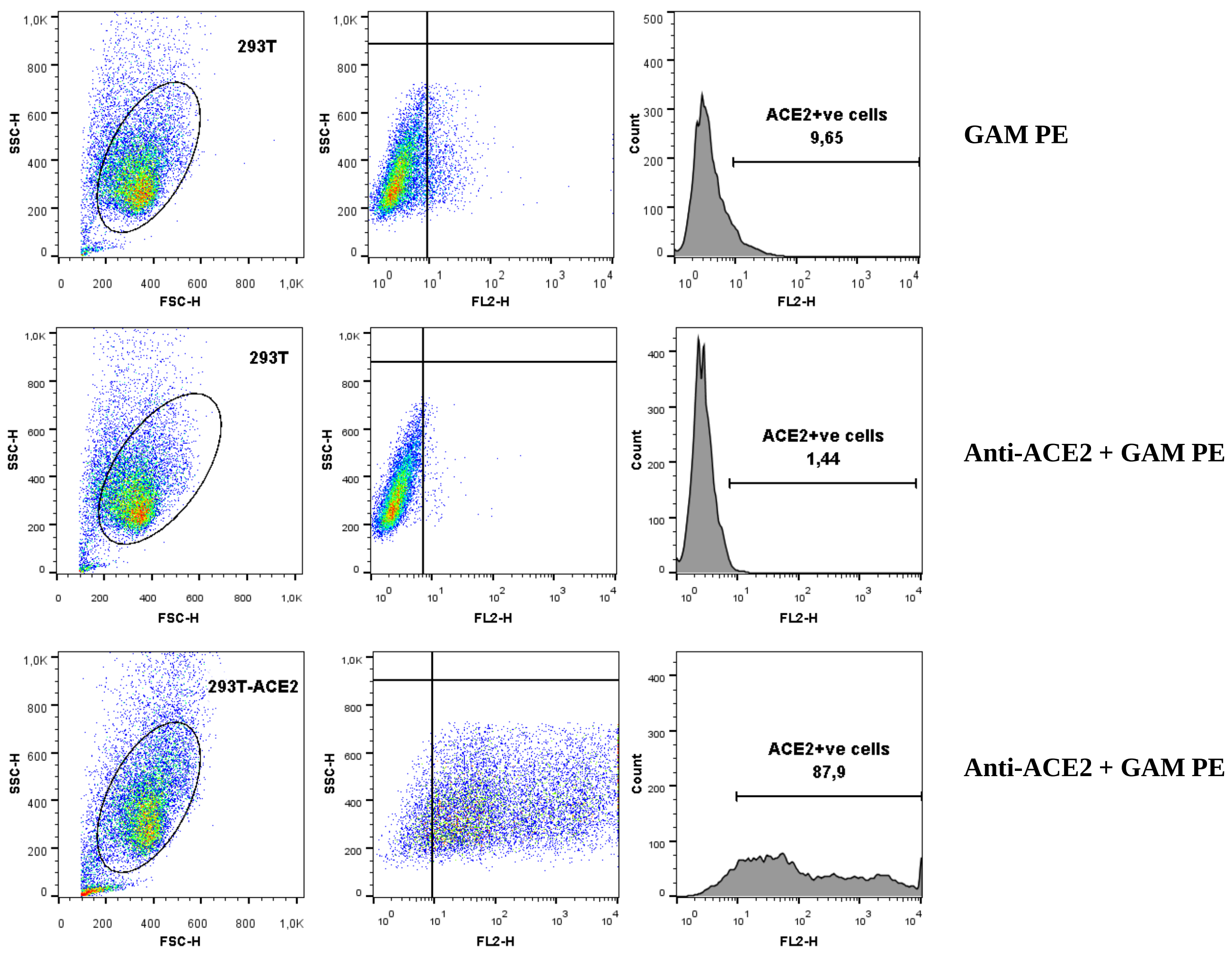

Supplementary Figure 3 . Human ACE2 expression in 293T Lenti-X cells. The identification of ACE2 positive cells was performed by using a primary mouse anti-human ACE2 antibody (Millipore, Catalog Number: MAB5676) followed by a secondary goat anti-mouse IgG-PE (SoutherBiotech; Catalog Number: 1030-09). Staining with secondary antibody only (goat anti-mouse, GAM PE) was used as negative control to set the gate of negative cells and quantify the percentage of positive cells expressing ACE2. 293T cells transfected with ACE2 expressing plasmid (phACE2 plasmid from Addgene, Cat# 1786) were used as positive control (293T-ACE2). The number within the histogram plot indicates the % of ACE2 positive cells.

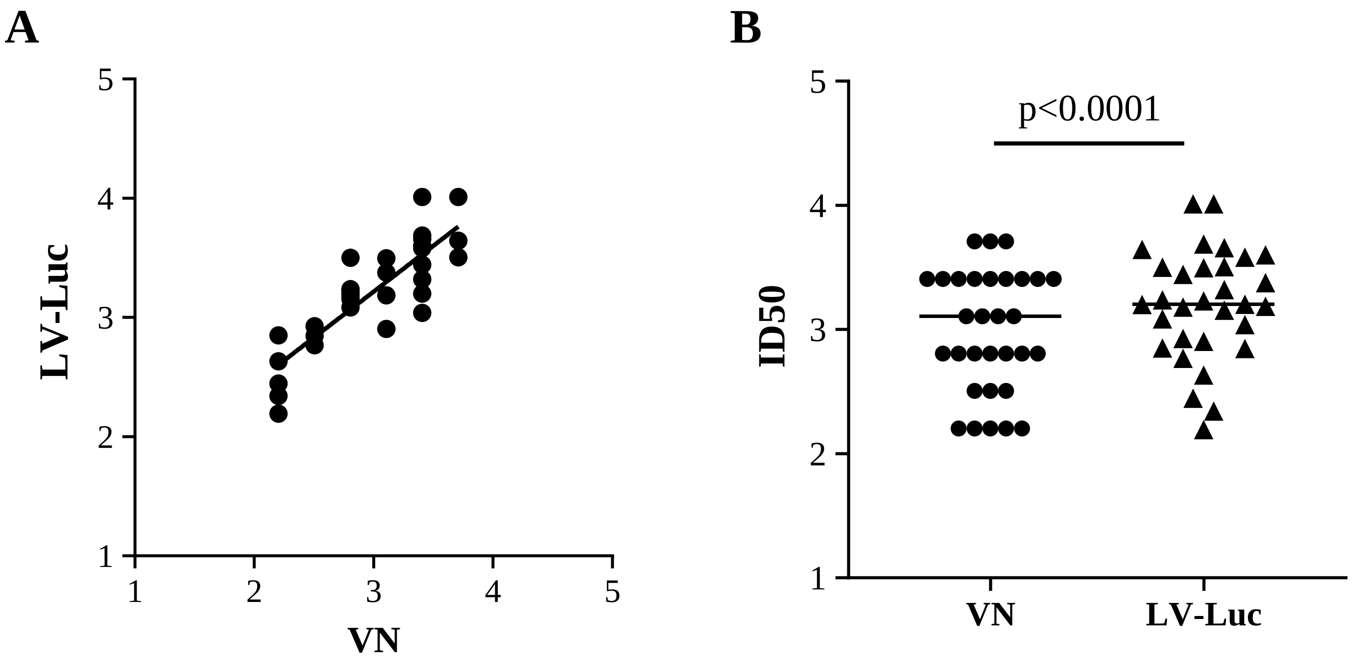

test	Spearman	Pearson
r	0,8344	0,8486
95% CI	0,6757-0,9192	0,7068-0,9249
p Value (two-tailed)	<0,0001	<0,0001

Supplementary Figure 4. Correlation between neutralization assays. Serum samples (N=31) collected from immunized animals were assayed using neutralization assays based on infectious SARS-CoV-2 (VN) and on lentiviral vector pseudotyped with Spike (LV-Luc). (**A**) Correlation between the ID50 (log10) values obtained from the two assays. Dots correspond to individual measurements; the black line represents the regression line. The table below shows the statistical analysis based on Spearman and Pearson correlation. (**B**) Comparison of ID50 (log10) values obtained using the two assays. A Wilcoxon matched-pairs signed rank test was used to compare the assays. The black line indicates the median ID50.

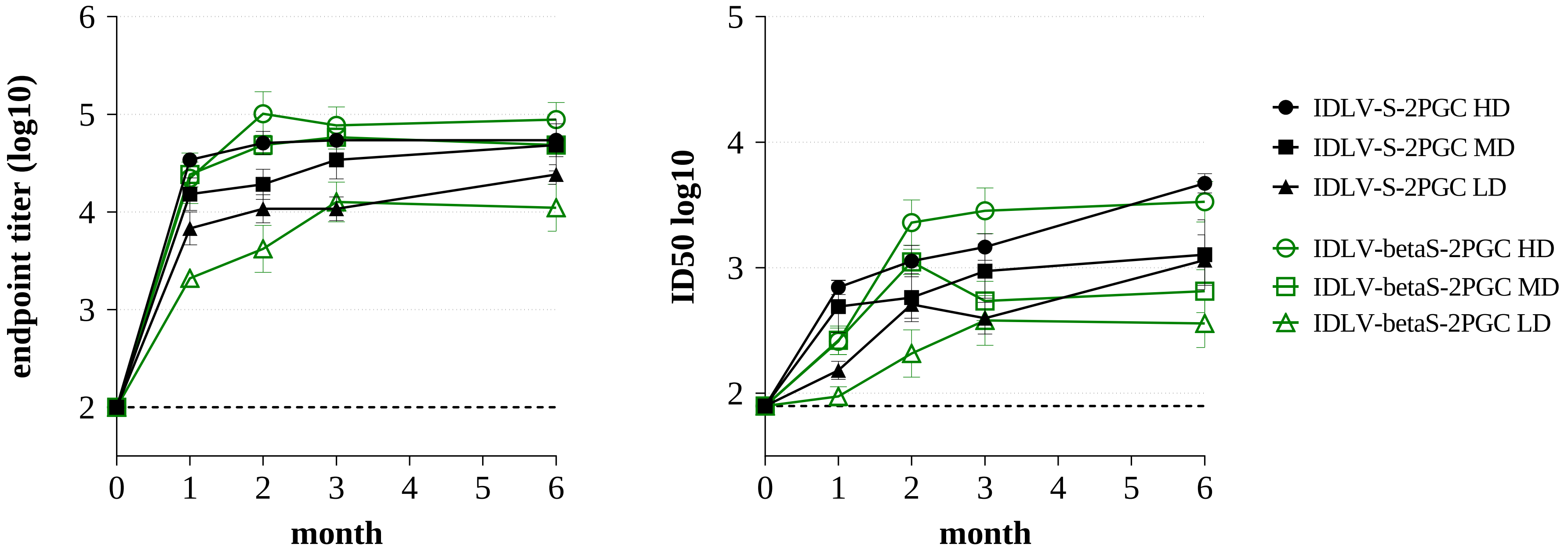

Supplementary Figure 5. Comparison between IDLV vaccines expressing Wuhan or Beta Spike. Kinetics of anti-RBD binding Abs (left panel) and anti-Wuhan nAbs (right panel) in mice immunized with IDLV-S-2PGC (black) and IDLV-betaS-2PGC (green).

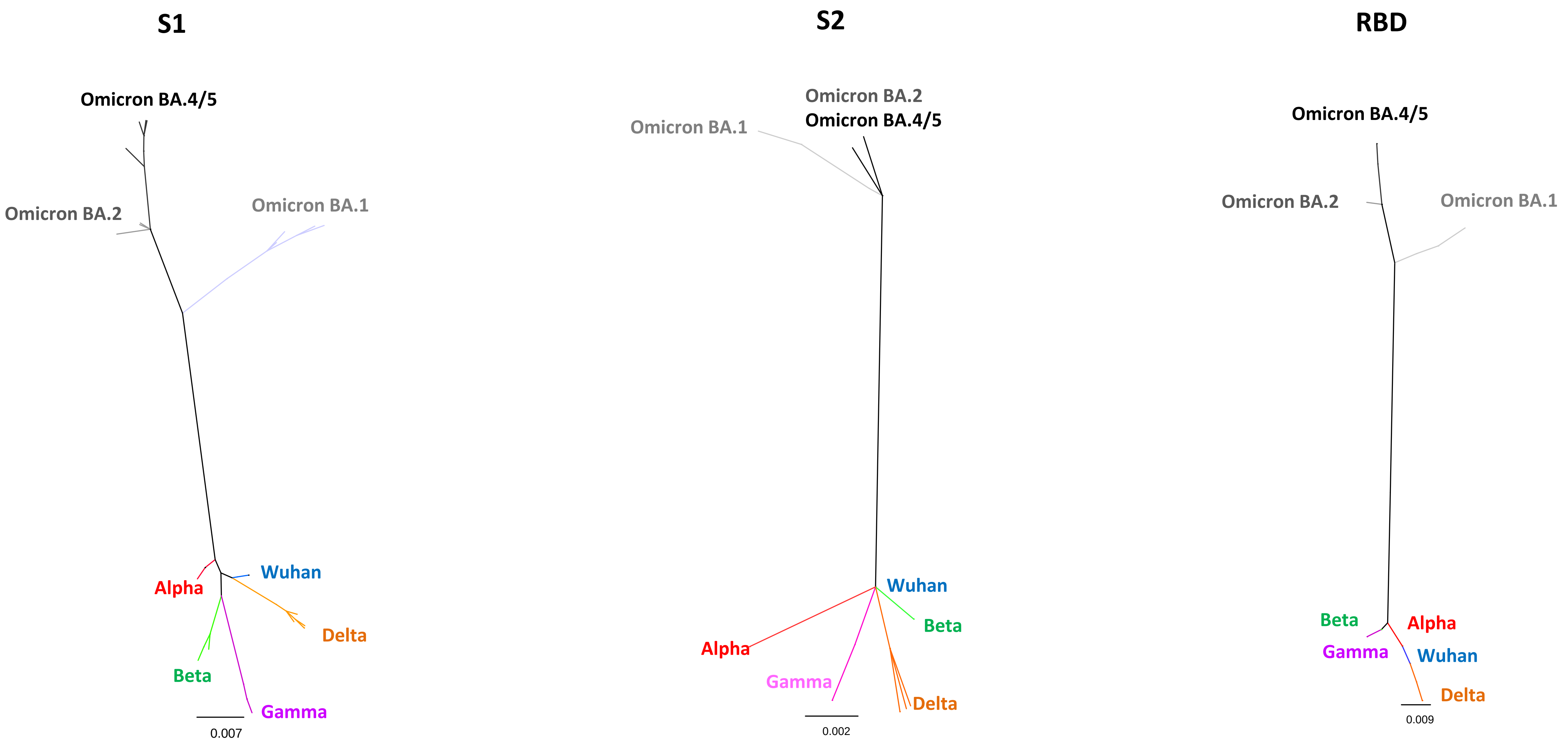

Supplementary Figure 6. Maximum likelihood phylogenetic tree inferred from S1, S2 and RBD amino acid sequences. The ancestral SARS-CoV-2 sequence (Wuhan-Hu-1), the Alpha, Beta, Gamma, Delta and the Omicron (BA.1, BA.2, BA.4/5) variants are highlighted with different colors. The scale bar at the bottom of the tree correspond to amino acid substitutions per site.

Supplementary Table 1.

We gratefully acknowledge the authors, originating and submitting laboratories of the genetic sequences and metadata made available through GISAID on which this research is based.

GISAID EpiCoV accession(s)	Originating laboratory	Submitting laboratory	Authors
**EPI_ISL_14471503**	Max von Pettenkofer Institute, Virology, National Reference Center for Retroviruses, LMU Munich	Laboratory for Functional Genome Analysis; Dept. Genomics; Gene Center of the LMU Munich	Max Muenchhoff; Stefan Krebs; Alexander Graf; Oliver Keppler; Helmut Blum
**EPI_ISL_14616786**	Biogroup-Labo-Biolam	Laborizon Centre	Julien Baillus, Christian Chillou, Alyssia Francois, Friconnet Marion, Eve Haguenoer, Anne Holstein, Mélanie Jimenez, Karolina Modzelewska, Claire Vignault, Stephane Watt
**EPI_ISL_14672772**	KU Leuven, Rega Institute, Clinical and Epidemiological Virology	KU Leuven, Rega Institute, Clinical and Epidemiological Virology	Tony Wawina-Bokalanga, Anne-Sophie Logist, Bram Van Holm, Robbe Sinnesael, Jens Verlinden, Levi Ysebaert, Bert Vanmechelen, Piet Maes
**EPI_ISL_15108686**	Servicio de Microbiologia, Hospital Puerta del Mar, Cadiz, Spain	Clinical Bioinformatics Area, Fundación Progreso y Salud	María Lara on behalf of SARS-CoV-2 whole genome sequencing circuit of Andalusia
**EPI_ISL_15165139**	UMC Groningen, Clinical Virology, Department of Medical Microbiology and Infection Prevention	UMC Groningen, Clinical Virology, Department of Medical Microbiology and Infection Prevention	A. Tami, I. van der Gun, A. C. M. Veloo, K. Wold, G. de Boer, M. Vincenti-Gonzalez, E. F. Lizarazo Forero, D. Pantano, M. van der Meer, A. Friedrich, B. Niesters
**EPI_ISL_15291623**	Dept Virology & Microbiological Special Diagnostics, Statens Serum Institut	Dept Virology & Microbiological Special Diagnostics, Statens Serum Institut	Polacek, C.

**EPI_ISL_11230221**	Austrian Agency for Health and Food Safety (AGES)	Bergthaler laboratory, CeMM Research Center for Molecular Medicine of the Austrian Academy of Sciences	Lukas Endler, Anna Schedl, Fabian Amman, Petr Triska, Matthew Thornton, Thomas Penz, Benedikt Agerer, Michelle Chan, Michael Schuster, Bekir Erguner, Jan Laine, Martin Senekowitsch, Christoph Bock, Andreas Bergthaler
**EPI_ISL_12655397**	Pharmgenetix GmbH	Pharmgenetix GmbH	S. Vanoni, A. Matulevicius, B. Avdiu, G. Scantamburlo, C. Nofziger
**EPI_ISL_12064979**	Centre Hospitalier Universitaire (CHU) Poitiers	Centre Hospitalier Universitaire (CHU) Poitiers	Luc Deroche, Manon Prat, Jean Philippe Da Mota, Magali Garcia, Agnes Beby-Defaux, Nicolas Leveque
**EPI_ISL_12516479**	Labor ZOTZ|KLIMAS; MVZ Düsseldorf-Centrum	Robert Koch Institute	Drechsel, Oliver
**EPI_ISL_13567182**	Rosalind Franklin Laboratory	Wellcome Sanger Institute for the COVID-19 Genomics UK (COG-UK) Consortium	Donald Fraser, Suki Lee, Rob Howes, The Rosalind Franklin Laboratory and Alex Alderton, Roberto Amato, Jeffrey Barrett, Sonia Goncalves, Ewan Harrison, David K. Jackson, Ian Johnston, Dominic Kwiatkowski, Cordelia Langford, John Sillitoe on behalf of the Wellcome Sanger Institute COVID-19 Surveillance Team
**EPI_ISL_13628374**	Pauls Stradiņš Clinical University Hospital	Riga East Clinical University Hospital, National Microbiology Reference Laboratory	Reinis Vangravs, Ģirts Šķenders, Jevgenijs Bodrenko, Kristīne Liepiņa, Anastasija Žuravļova, Arzu Algulieva, Reinis Zeltmatis, Dārta Pūpola, Jūlija Čevere, Oksana

			Savicka, Ilva Pole, Diāna Dušacka, Sergejs Ņikišins, Jānis Meisters, Zane Dobele
**EPI_ISL_13951654**	National Virus Reference Laboratory	National Virus Reference Laboratory	Zoe Yandle, Charlene Bennett, Gabriel Gonzalez, Michael Carr, Jonathan Dean, Cillian F De Gascun
**EPI_ISL_15141596**	Biogroup Laboratoire Biorylis Saint Gilles	Laborizon Centre	Julien Baillus, Christian Chillou, Alyssia Francois, Friconnet Marion, Eve Haguenoer, Anne Holstein, Mélanie Jimenez, Karolina Modzelewska, Claire Vignault, Stephane Watt
**EPI_ISL_15147441**	Robert Koch-Institut ZBS1 (Zentrum für biologische Gefahren und spezielle Pathogene hochpathogene Viren)	Robert Koch Institute	Drechsel, Oliver
**EPI_ISL_15153853**	Lifebrain Covid Labor GmbH	Lifebrain Covid Labor GmbH	Filip Sima, Alexandra Wagner, Kristina Bavrka Kolenc, Lucia Castello, Sojung Han, Anna Edermayr
**EPI_ISL_15211054**	National Public Health Organization	16672 Vari, Athens	Tryfinopoulou, Kyriaki
**EPI_ISL_15269080**	Laboratoire De Soleau	Laborizon Centre	Julien Baillus, Christian Chillou, Alyssia Francois, Friconnet Marion, Eve Haguenoer, Anne Holstein, Mélanie Jimenez, Karolina Modzelewska, Claire Vignault, Stephane Watt
**EPI_ISL_15287572**	Servicio de Microbiología. Hospital Clínico Universitario de Valencia	FISABIO_DGSP_COVIDSurveillance	David Navarro Ortega, Eliseo Albert Vicent, Ignacio Torres and Consortium for Genomic Surveillance of SARS-CoV-2 in Comunitat Valenciana
**EPI_ISL_14772138**	Respiratory Virus Unit,	COVID-19 Genomics UK (COG-UK) Consortium	PHE Covid Sequencing Team

	Microbiology Services Colindale, Public Health England		
**EPI_ISL_14951688**	Labo Analyses Med	National Reference Center for Viruses of Respiratory Infections, Institut Pasteur, Paris	Marion Barbet, Méline Bizard, Angela Brisebarre, Camille Capel, Vincent Enouf, Louise Lefrançois, Frédéric Lemoine, Christophe Malabat, Corinne Maufrais, Slim El Khiari, Julien Fumey, Etienne Simon-Lorière, Maud Vanpeene, Sylvie Van der Werf, David ROSSIGNOL
**EPI_ISL_15030352**	Servicio de Microbiología. Hospital General Universitario de Castellón	FISABIO_DGSP_COVIDSurveillance	Marta Gil Barrachina, María Dolores Tirado Balaguer and Consortium for Genomic Surveillance of SARS-CoV-2 in Comunitat Valenciana
**EPI_ISL_15038833**	Institute of Microbiology and Immunology, Faculty of Medicine, University of Ljubljana	Institute of Microbiology and Immunology, Faculty of Medicine, University of Ljubljana	Alen Suljič, Samo Zakotnik, Tomaž Mark Zorec, Doroteja Vlaj, Tina Živič, Tina Gabrovšek, Patricija Pozvek, Špela Pleh, Miša Korva, Mario Poljak, Tatjana Avšič - Županc
**EPI_ISL_15072165**	Respiratory Virus Unit, Microbiology Services Colindale, Public Health England	COVID-19 Genomics UK (COG-UK) Consortium	PHE Covid Sequencing Team
**EPI_ISL_15111587**	Labo Analyses Med	National Reference Center for Viruses of Respiratory Infections, Institut Pasteur, Paris	Marion Barbet, Méline Bizard, Angela Brisebarre, Camille Capel, Vincent Enouf, Louise Lefrançois, Frédéric Lemoine, Christophe Malabat, Corinne Maufrais, Samar Berreira, Slim El Khiari, Etienne Simon-Lorière, Maud

			Vanpeene, Sylvie Van der Werf, Sophie ZAFFREYA
**EPI_ISL_15149214**	Limbach - MVZ Humangenetik Ulm	Robert Koch Institute	Drechsel, Oliver
**EPI_ISL_15287581**	Servicio de Microbiología. Hospital Clínico Universitario de Valencia	FISABIO_DGSP_COVIDSurveillance	David Navarro Ortega, Eliseo Albert Vicent, Ignacio Torres and Consortium for Genomic Surveillance of SARS-CoV-2 in Comunitat Valenciana
**EPI_ISL_14976943**	WSSE Kielce	Wojewodzka Stacja Sanitarno-Epidemiologiczna w Rzeszowie, Laboratorium Diagnostyki Medycznej	Marzena Baranowska, Katarzyna Wilk, Karolina Ostrowska, Anna Nowakowska
**EPI_ISL_15140433**	EUC Inc.	National Institute of Public Health	Helena Jirincova, Jaromira Vecerova, Timotej Suri, Jan Moskalyk, Alexander Nagy
**EPI_ISL_15147701**	Labor ZOTZ|KLIMAS; MVZ Düsseldorf-Centrum	Robert Koch Institute	Drechsel, Oliver
**EPI_ISL_15195935**	Labo Analyses Med	National Reference Center for Viruses of Respiratory Infections, Institut Pasteur, Paris	Marion Barbet, Méline Bizard, Angela Brisebarre, Camille Capel, Vincent Enouf, Louise Lefrançois, Frédéric Lemoine, Christophe Malabat, Corinne Maufrais, Samar Berreira, Slim El Khiari, Etienne Simon-Lorière, Maud Vanpeene, Sylvie Van der Werf, Beate HEYM
**EPI_ISL_15208571**	Department of Bacteria, Parasites and Fungi, Statens Serum Institut, Copenhagen, Denmark	Statens Serum Institut Bioinformatics and Microbial Genomics	Danish Covid-19 Genome Consortium
**EPI_ISL_15212518**	Respiratory Virus Unit, Microbiology Services Colindale,	COVID-19 Genomics UK (COG-UK) Consortium	PHE Covid Sequencing Team

	Public Health England		
**EPI_ISL_15216058**	Dutch COVID-19 response team	National Institute for Public Health and the Environment (RIVM)	Adam Meijer, Harry Vennema, Dirk Eggink, Jeroen Cremer, Sharon van den Brink, Bas van der Veer, AnneMarie van den Brandt, Lisa Wijsman, Kim Freriks, Ryanne Jaarsma, Lynn Aarts, Sanne Bos, Jil Kocken, Jordy de Bakker, Afke Vogelzang, Hai Xi Wiegmans, Florian Zwagemaker, Dennis Schmitz, Annelies Kroneman, Karim Hajji, Ivo van Walle, Chantal Reusken, on behalf of the national COVID-19 response team
**EPI_ISL_13283028**	AZ Sint-Jan Brugge-Oostende AV	Sciensano, Viral Diseases Laboratory	François Dufrasne, Reinout Van Eycken, Bert Monsieurs, Michael Peeters, Marijke Renders, Steven Van Gucht, Cyril Barbezange, Sarah Denayer
**EPI_ISL_14949215**	Paris - Saint Antoine - SMIT	IAME UMR1137 Inserm, Université Paris Cité, Hôpital Bichat	Antoine Bridier-Nahmias, Romain Coppée, Matilda Berkell, Samuel Lebourgeois, Surbhi Malhotra, Anna Maria Franco Yusti, Quentin Le Hingrat, Lena Daniel, Gilles Collin, Alexandre Storto, Mélanie Bertine, Charlotte Charpentier, Nadhira Houhou-Fidouh, Diane Descamps, Benoit Bisseaux
**EPI_ISL_15137335**	Labor Dr. Heidrich & Kollegen MVZ GmbH Hamburg	Robert Koch Institute	Drechsel, Oliver

**EPI_ISL_5069977**	Dr. Risch Ostschweiz AG	Microbiology	Nadia Wohlwend, Faina Wehrli, Dominique Fabien Hilti, Sinem Kas, Martin Risch, Thomas Bodmer, Lorenz Risch
**EPI_ISL_6230389**	Dutch COVID-19 response team	National Institute for Public Health and the Environment (RIVM)	Adam Meijer, Harry Vennema, Dirk Eggink, Jeroen Cremer, Sharon van den Brink, Bas van der Veer, AnneMarie van den Brandt, Lisa Wijsman, Kim Freriks, Ryanne Jaarsma, Euníce Then, Lynn Aarts, Sanne Bos, Stijn van Rossum, Florian Zwagemaker, Dennis Schmitz, Annelies Kroneman, Karim Hajji, Ivo van Walle, Chantal Reusken, on behalf of the national COVID-19 response team
**EPI_ISL_7806545**	MEPHI, Aix Marseille University	MEPHI, Aix Marseille University	Anthony LEVASSEUR
**EPI_ISL_10631767**	Respiratory Virus Unit, Microbiology Services Colindale, Public Health England	COVID-19 Genomics UK (COG-UK) Consortium	PHE Covid Sequencing Team
**EPI_ISL_14022807**	Regional Virus Laboratory, Belfast Health and Social Care Trust; and: Genomics Core Technology Unit, Queen's University Belfast.	COVID-19 Genomics UK (COG-UK) Consortium	[Regional Virus Laboratory, BHSCT]: Conall McCaughey, James McKenna, Tanya Curran, Susan Feeney, Alison Watt, Ciara Cox, Mairead Connor, Zoltan Molnar, David Simpson, Derek Fairley; [Genomics Core Technology Unit, QUB]: Marc Fuchs, Clara Radulescu, Miao Tang, Arun Mahesh, Deborah Lavin, Syed Umbreen, Sarah

			Sonner, Jana Gazdova, Evan Troendle, Alan, Rice, Timofey Skvortsov, Fiona Rogan, Julia Miskelly, Stephen Bridgett, David Simpson
**EPI_ISL_14095562**	Red de Vigilancia Genómica para la COVID-19 en Canarias	División de Genómica, ITER	Laura Ciuffreda; Rafaela González-Montelongo; Julia Alcoba-Florez; Diego García-Martínez de Artola; Helena Gil-Campesino; Hector Rodriguez-Perez; Antonio Iñigo-Campos; Isabel De Miguel-Martínez; Tomas Tosco-Nuñez; Oscar Diez-Gil; Agustin Valenzuela-Fernandez; Jose Miguel Lorenzo-Salazar; Carlos Flores
**EPI_ISL_14616814**	Biogroup-Labo-Biolam	Laborizon Centre	Julien Baillus, Christian Chillou, Alyssia Francois, Friconnet Marion, Eve Haguenoer, Anne Holstein, Mélanie Jimenez, Karolina Modzelewska, Claire Vignault, Stephane Watt
**EPI_ISL_6115483**	INSA	Instituto Nacional de Saude (INSA)	Borges et al
**EPI_ISL_6375258**	Karolinska University Hospital Solna	Karolinska University Hospital	Jan Albert ,Tobias Allander ,Annelie Bjerkner ,Sandra Broddesson ,Robert Dyrdak ,Martin Ekman ,Lynda Eneh ,Lina Guerra Blomqvist ,Karolina Ininbergs ,Tanja Normark ,Isak Sylvin ,Zhibing Yun ,Martina Wahlund ,Valtteri Wirta
**EPI_ISL_8765357**	Tyrolpath Obrist Brunhuber GmbH	Tyrolpath Obrist Brunhuber GmbH	Clemens Mayer, Samuel Huter, Gerold Untergasser, Lisa Eiterer, Manuel Stampfer, Chiara Ennemoser, Janine

			Kraussler, Roland Werner, Philipp Knabl, Yasemin Caf, Peter Obrist, Ludwig Knabl
**EPI_ISL_2017663**	CLILAB	Microbiology Department	Sara Marti, Aida Gonzalez-Diaz, Laura Calatayud, Jordi Niubó, Miguel Fernandez-Huerta, Carmen Ardanuy, Jordi Camara, M Angeles Domínguez
**EPI_ISL_2047772**	Hospital Universitari Vall d'Hebron - Vall d'Hebron Institut de Recerca	Hospital Universitari Vall d'Hebron - Vall d'Hebron Institut de Recerca	Cristina Andrés, Maria Piñana, Damir Garcia-Cehic, Ariadna Rando, Juliana Esperalba, Maria Gema Codina, Carla Castillo, Maria Carmen Martin, Tomàs Pumarola, Josep Quer, Andrés Antón
**EPI_ISL_2281368**	Microbiology Department, Laboratori Clínic Metropolitana Nord. Hospital Universitari Germans Trias i Pujol	Can Ruti SARS-CoV-2 Sequencing Hub (HUGTiP/IrsiCaixa/IGTP)	Marc Noguera-Julian, Pilar Armengol, Ignacio Blanco, Antoni E Bordoy, Francesc Catala-Moll, Pere-Joan Cardona, Maria Casadellà, Cristina Casañ, Gemma Clara, Bonaventura Clotet, Cristina Esteban, Montserrat Giménez, Mercedes Guerrero, Anna Not, Roger Paredes, Mariona Parera, Verónica Saludes, Alba Sánchez, and Elisa Martró on behalf of the Can Ruti SARS-CoV-2 Sequencing Hub.
**EPI_ISL_12655397**	Pharmgenetix GmbH	Pharmgenetix GmbH	S. Vanoni, A. Matulevicius, B. Avdiu, G. Scantamburlo, C. Nofziger
